# Dramatic Effect of Alkali Metal Alkoxides on the Anionic Copolymerization of Styrene and Isoprene

**DOI:** 10.1021/acs.macromol.5c00975

**Published:** 2025-06-16

**Authors:** Dominik A. H. Fuchs, Holger Frey, Axel H. E. Müller

**Affiliations:** Department of Chemistry, 9182 Johannes Gutenberg University Mainz, Duesbergweg 10-14, Mainz 55128, Germany

## Abstract

The effect of lithium, sodium, and potassium *tert*-amylates on the kinetics of the statistical anionic copolymerization of styrene and isoprene in cyclohexane was investigated using *in situ* near-infrared (NIR) spectroscopy. The reactivity ratios and the related comonomer gradients can be adjusted over the entire range resulting in both random and inverted gradient copolymers. Lithium *tert*-amylate retards the polymerization at overstoichiometric concentrations. In contrast, even at low concentrations, sodium and potassium *tert*-amylate increase the rate of styrene polymerization due to a counterion exchange. Only 1/30 equiv of potassium *tert*-amylate relative to butyllithium is necessary to obtain random copolymers, which unexpectedly consist of short blocks. Remarkably, a high content of isoprene 1,4-units is maintained, leading to a low glass transition temperature of −55 °C of random or inversely tapered poly­(styrene*-co-*isoprene). Thus, in contrast to Lewis base modifiers, the diene microstructure can be decoupled from reaction kinetics, when potassium alkoxides are used.

## Graphical abstract

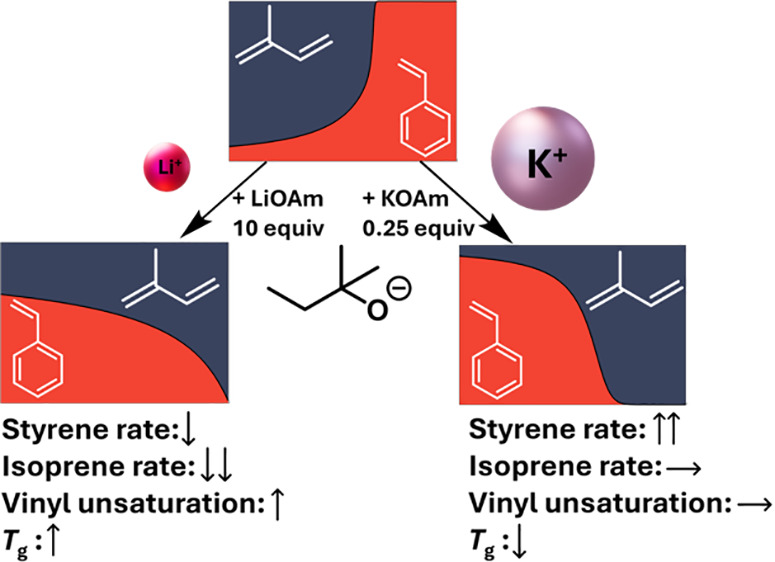

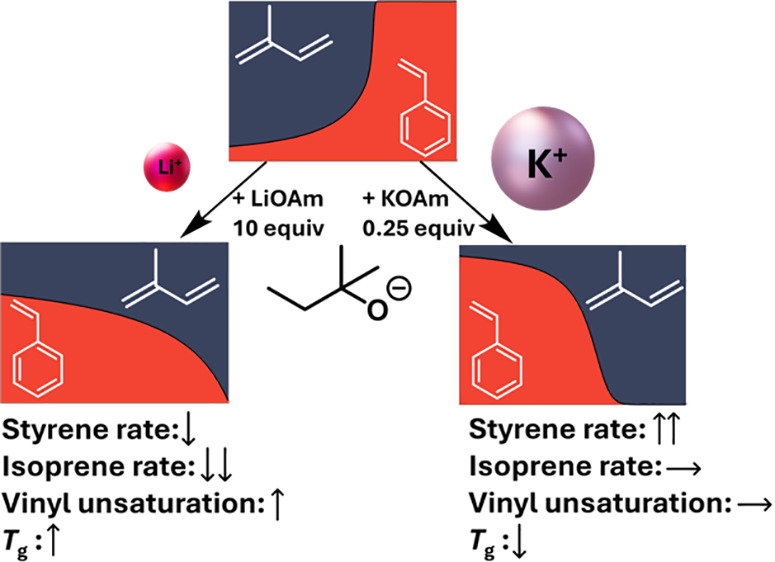

## Introduction

Living anionic polymerization, discovered by Szwarc in 1956, enables the synthesis of a wide range of well-defined (multi) block copolymers.^1^ Although this polymerization technique places high demands on the purity of both monomers and solvents, it has been used for the synthesis of thermoplastic elastomers (TPEs) for a long time.^2,3^ These phase-separated materials consist of at least three different blocks in an ABA structure, where polymer A is a block with a high glass transition temperature, *T*_g_, usually styrene (S), and the midblock B is a highly flexible polymer with a low *T*_g_, usually a poly­(1,3-diene), commonly based on butadiene (B) or isoprene (I).^4–6^ A large variety of products are synthesized from the combination of styrene and diene by changing polymer architecture, composition, molecular weight, and microstructure of the diene. Biobased and specialized monomers have also been used to synthesize (block)­copolymers with tailored properties for high-performance materials.^5–13^ Here, block copolymers are synthesized by stepwise addition of individual monomers.

Very early, it was discovered that the statistical copolymerization of styrene and dienes initiated by butyllithium (BuLi) in nonpolar solvents leads to “tapered” copolymers displaying similar properties as block copolymers.^2,3^ In these solvents, the diene polymerizes first, and most of the styrene is incorporated after complete consumption of the diene. This translates to disparate reactivity ratios *r*_S_ = 0.05; *r*_B_ = 15 and *r*_S_ = 0.013; and *r*_I_ = 10.1 for the S/B^14,15^ and S/I^16^ system, respectively, resulting in strong gradient copolymers.^2,3^ The reason for these strong differences in the reactivity ratios is the large discrepancies in the crossover rates, *k*_SI_ ≫ *k*_IS_.^16,17^

In apolar solvents, such as cyclohexane, the living, lithiated polymer chain ends form inactive dimers in equilibrium with the nonaggregated chains, acting as polymerization centers (Scheme 1).^14,15,19–22^ The addition of polar additives, so-called modifiers, breaks up the aggregates and increases the reactivity of the comonomers to different extents. Typical modifiers are Lewis bases like methyl-*tert*-butylether (MTBE), THF, 2,2-di­(2-tetrahydrofuryl)­propane (DTHFP), or amines like tetramethylethylenediamine (TMEDA).^23–25^ By using increasing equivalents (equiv) of these modifiers with respect to the butyllithium initiator, the reactivity ratios converge, leading to random copolymers or even inverted gradients.^18,23,26,27^ However, the use of these polar modifiers leads to a strong decrease of 1,4-microstructures in the polydiene, thus increasing the glass transition temperature, which limits their usage as thermoplastic elastomers.^26,28–30^

**Scheme 1:**
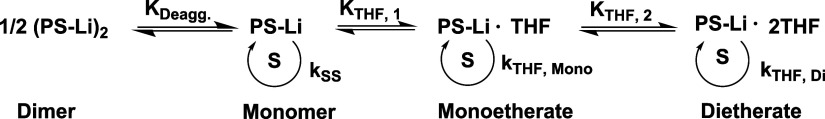
Aggregation of Polystyryllithium in Apolar Solvents and the Effect of Lewis Bases (Example: THF) on the Aggregation Equilibrium^a^

a Reproduced from ref 17 with permit from the American Chemical Society.

In previous works, several authors have investigated the effects of Lewis acid ligands, and specifically alkali alkoxides (Li, Na, K, Rb, Cs), on the homo- and copolymerization of styrene and butadiene.^31–37^ These highly aggregated modifiers form mixed aggregates with the polymer chain end, changing reactivity.^34,38^ Addition of 6 equiv lithium *tert*-butoxide (LiOtBu) relative to butyllithium reduced the homopolymerization rate by a factor of 6.25 for butadiene and 2.3 for styrene.^33^

The introduction of higher alkali alkoxides results in an intermolecular exchange of counterions via mixed aggregates (Scheme 2). This equilibrium must be fast, as low dispersities (*Đ* < 1.1) and the desired molecular weights were achieved.^31,37,39–43^ However, different authors disagree on the question whether the mixed aggregate is active in polymerization.^44–46^ As a general trend, increasing amounts of these modifiers and increasing counterion size increase the homopolymerization rate of both styrene and diene monomers, and styrene is more accelerated than the diene.^31,47^

**Scheme 2:**
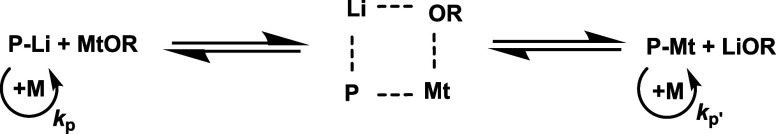
Two-State Model of Polymerization^a^

a Adapted with permission from ref 31. Copyright 1969, John Wiley and Sons.

This influence does not only affect kinetics but also determines the microstructure of the polydiene and, consequently, the glass transition temperature. Literature results on the vinyl unsaturation of polybutadiene in the presence of an excess of lithium alkoxides scatter widely. Makowski recorded an increase up to 40% vinyl unsaturation, while Hsieh only found an increase to 10%.^33,48^ In contrast, the addition of 1 equiv sodium *tert*-butoxide (NaO*t*Bu) already increases the vinyl unsaturation of polybutadiene from 5 to 70%,^31,49,50^ which is the microstructure obtained with metallic or organosodium initiators.^36,49,51–53^ Overall, sodium alkoxides exert the strongest modifier effect on the microstructure.^49^

Various groups investigated the effect of potassium alkoxides in sub- and overstoichiometric concentrations for the polymerization of butadiene and isoprene on their respective microstructure.^31,41,47,54^ Depending on the modifier concentration, a wide range of vinyl unsaturations of 20 to 50% was observed. Both metallic and organopotassium initiators also increased vinyl unsaturation to 50%.^49,52,53,55^ Significant differences in the vinyl content were observed between butadiene and isoprene at comparable modifier concentrations,^32,47^ which was explained by the PI/KOtBu adduct acting as a Schlosser-Lochmann base, deprotonating the 2-methyl group.^41,47,56–58^ The higher alkali alkoxides of rubidium and cesium qualitatively had similar effects as potassium.^32^

The statistical copolymerization of styrene and butadiene in the presence of various alkali alkoxides was investigated in several studies. In all studies, only the rate of total comonomer consumption and the fraction of styrene units in the copolymer as a function of conversion were determined, the latter being a rough estimate of the comonomer gradient along the chain.^32,33,59–61^ The addition of up to 6 equiv LiO*t*Bu retarded the reaction but had no significant impact on the gradient, which is quite surprising in view of the previous results of the homopolymerizations.^32,33^

Higher alkali alkoxides had strong effects on the copolymerization rate and on the styrene incorporation, which increased from Na ≪ K ∼ Rb < Cs.^32^ Already 0.2 equiv of NaO*t*Bu is sufficient to achieve random copolymerization.^32^ Organosodium initiators were also investigated and reactivity ratios determined as *r*_S_ = 0.42 and *r*_B_ = 0.3.^62^ These initiators undergo chain transfer to toluene, which can be suppressed by the addition of lithium alkoxides.^36^ It could be assumed that the main reaction center is the polymer anion with lithium as the counterion, but the high vinyl content of 70% is not consistent with this assumption.^31,49,50^ Therefore, it was concluded that the main reaction center is a bimetallic mixed complex (intermediate in Scheme 2), which differs from either of the initial compounds.^51,63^

The use of potassium alkoxides in the copolymerization of styrene and butadiene drastically changes the comonomer incorporation; already 1/30 equiv is sufficient for a random copolymerization, and at higher ratios (up to 1 equiv), inversion of the gradient was observed.^32,60,61,64,65^ Various publications^66,67^ and patents describe the use of potassium alkoxides for the synthesis of random S/B copolymers.^68–71^ Wofford and Hsieh as well as Arest-Yakubovich and co-workers studied the copolymerization of styrene and butadiene in cyclohexane at 25 °C with an organopotassium initiator and also found an inversion of reactivities compared to butyllithium (*r*_S_ = 3.3 and *r*_B_ = 0.12).^32,59^

The mechanism of homo- and copolymerization in the presence of sodium and potassium alkoxides is not fully understood. Two different mechanisms have been proposed,^37,72,73^ a two-state mechanism^32,45^ and a single-state mechanism consisting of a multicomponent complex, i.e., a mixed aggregate,^36,44,51^ as the reaction center, as shown in Scheme 2. The two-state mechanism assumes a reversible exchange of counterions; a mixed aggregate is not considered or is inactive. Each of the two propagating centers, when active, leads to its own microstructure.^32,39–43,73^

This work presents the first in-depth kinetic investigation on the copolymerization of styrene and isoprene in the presence of lithium, sodium, and potassium alkoxides in cyclohexane. Using *in situ* near-infrared (NIR) spectroscopy enabled us to independently track the conversion of both monomers and thus determine reactivity ratios and comonomer gradients along the polymer chain. Investigations on their microstructure, blockiness, and glass transition temperature enabled a better understanding of the polymerization mechanisms with Lewis acid modifiers.

## Experimental Section

Materials, instrumentation, and a general description of the copolymerization kinetic investigations are described in the Supporting Information.

## Results and Discussion

The kinetic effect of alkali metal alkoxides in the copolymerization of styrene and isoprene in cyclohexane was investigated using *in situ* near-infrared (NIR) spectroscopy at 20–23 °C. Lithium, sodium, and potassium *tert*-amylates served as modifiers. Deprotonated *tert*-amyl alcohol (2-methyl-2-butanolate) was used due to its solubility in cyclohexane, commercial availability, and industrial application.^60,64,69,70^ The initiator *sec-*butyllithium (BuLi) was added to a premixed solution of alkoxide and monomers to avoid side reactions (see Scheme S1).^41^ An equimolar monomer feed (*f*_S_ = *f*_I_ = 0.5) was used to generate a polymer with 60 wt % (57 vol %) of styrene. The targeted molecular weight of 80 kg/mol and low dispersities, Đ ≤ 1.1, were successfully achieved (Table 1 and Figures S5–S7). Higher molecular weights, which in some cases exceed theory, can be partially explained by an overestimation of the PS calibration, which is noted to be approximately 10%,^16^ and to a minor extent by termination reactions during initiation, as the alkoxides used cannot be dried further.

**Table 1:**
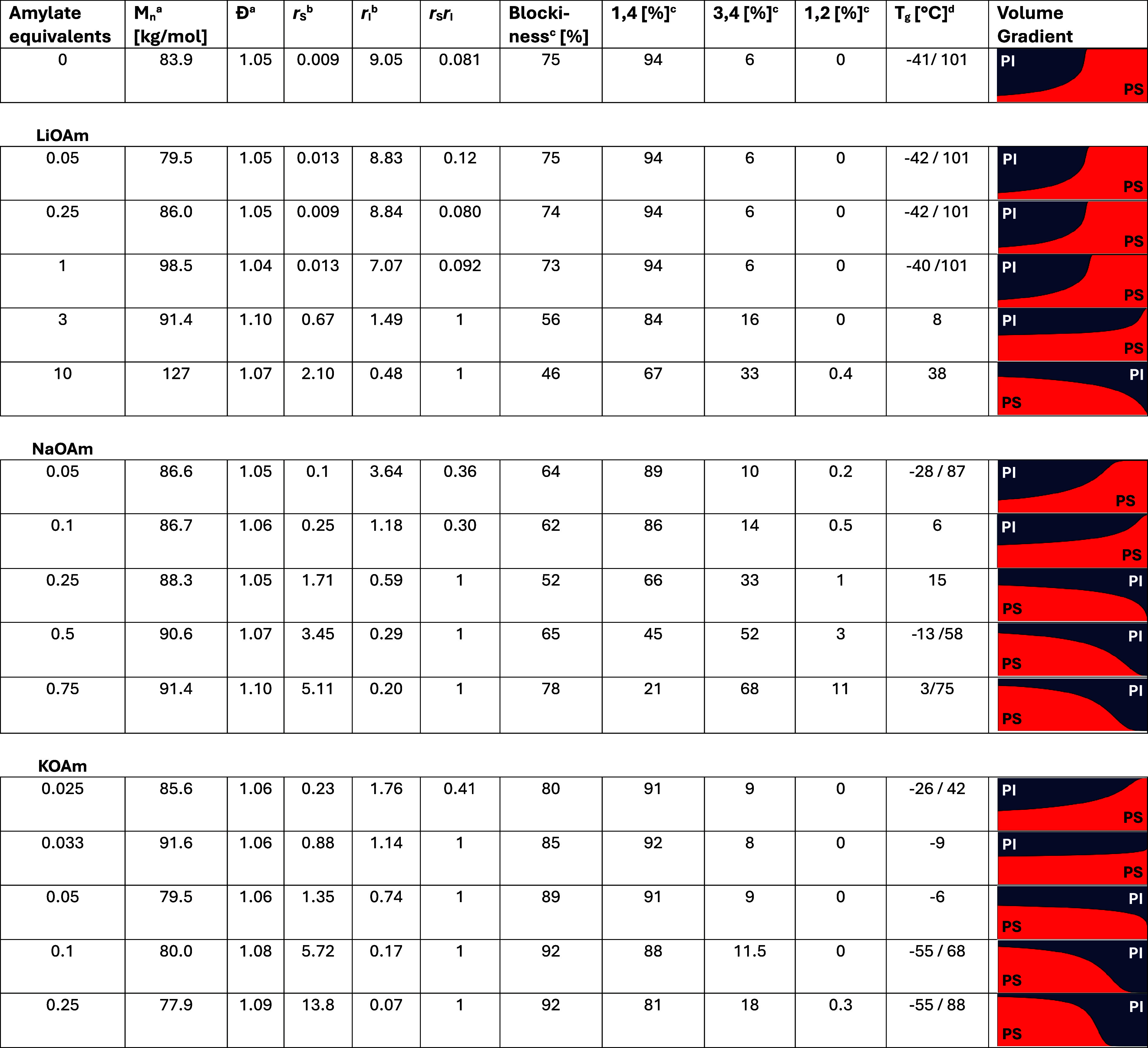
Effect of Alkali *tert-*Amylates on the Copolymerization of Styrene and Isoprene

a Determined by SEC with PS calibration.

b Calculated by either Jaacks or Meyer-Lowry.

c Determined by ^1^H NMR spectroscopy (see Supporting Information, Sections 4.2 and 5).

d Measured by DSC, value taken from the second heating cycle.

Time–conversion and individual versus total conversion plots for all counterions and all modifier concentrations are given in the Supporting Information in Figures S8–S13. The reactivity ratios were calculated according to the terminal model (Meyer-Lowry^74^ fit) or the nonterminal model (Jaacks^75,76^ fit); see Figures S15–S20. As stated in our previous publications,^8,16,17,23,24^ we assume validity of the nonterminal model (*r*_1_ *r*_2_ = 1) whenever the Jaacks plot is linear, in order to avoid overfitting.^77^ The Meyer-Lowry method was only used when the nonterminal model failed, i.e., for the copolymerization in pure cyclohexane^17,23,24^ and for small amounts of modifiers, [LiOAm]/[BuLi] ≤ 1; [NaOAm]/[BuLi] ≤ 0.1; [KOAm]/[BuLi] ≤ 0.025.

### Effect of Lithium *tert*-Amylate (LiOAm)

Lithium *tert*-amylate was specifically chosen to separate the effect of the introduced alkoxide from that of the added counterion. Up to an equimolar ratio of LiOAm to BuLi, no significant impact on the copolymerization kinetics is observed (Table 1, Figure 1, and Figures S8 and S9); only when the LiOAm content is increased to ≥3 equiv is the reaction is retarded and reactivity ratios change. At 10 equiv of LiOAm, the reaction slows down 7-fold for isoprene but less for styrene (for estimated half-lives; see Figure S14a). This is due to the formation of mixed aggregates (P–Li)_*x*_(LiOAm)_*y*_, decreasing the concentration of nonaggregated chain ends (Scheme 3).^78^ This effect is stronger for isoprene than that for styrene.

**Figure 1:**
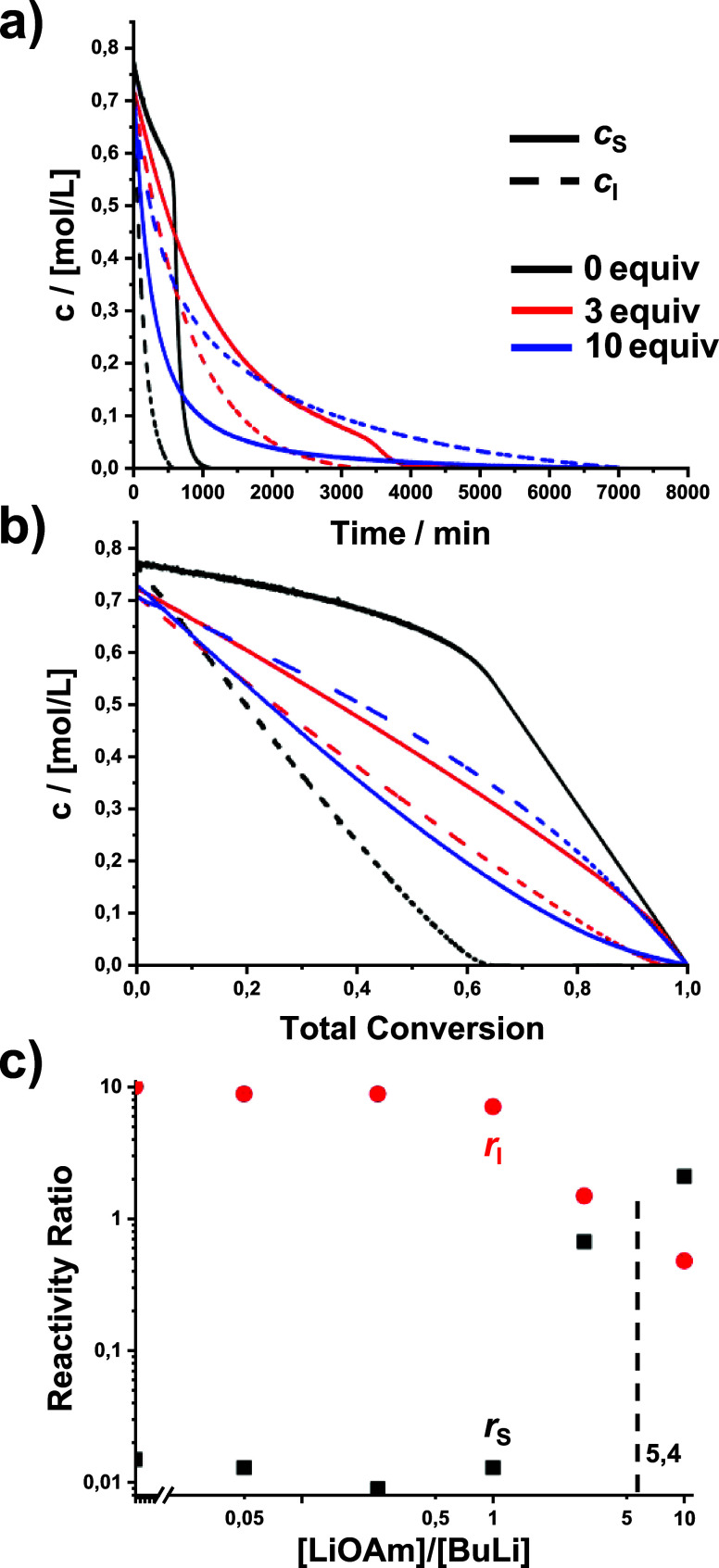
Effect of lithium *tert*-amylate (LiOAm) on the copolymerization kinetics: (a) time–conversion plots, (b) plots of comonomer concentrations versus total conversion, and (c) reactivity ratios. Red circles: isoprene, black squares: styrene. The dashed line indicates that *r*_S_ = *r*_I_ = 1 and thus (ideal) random copolymerization. Please note the double-logarithmic scales.

**Scheme 3:**
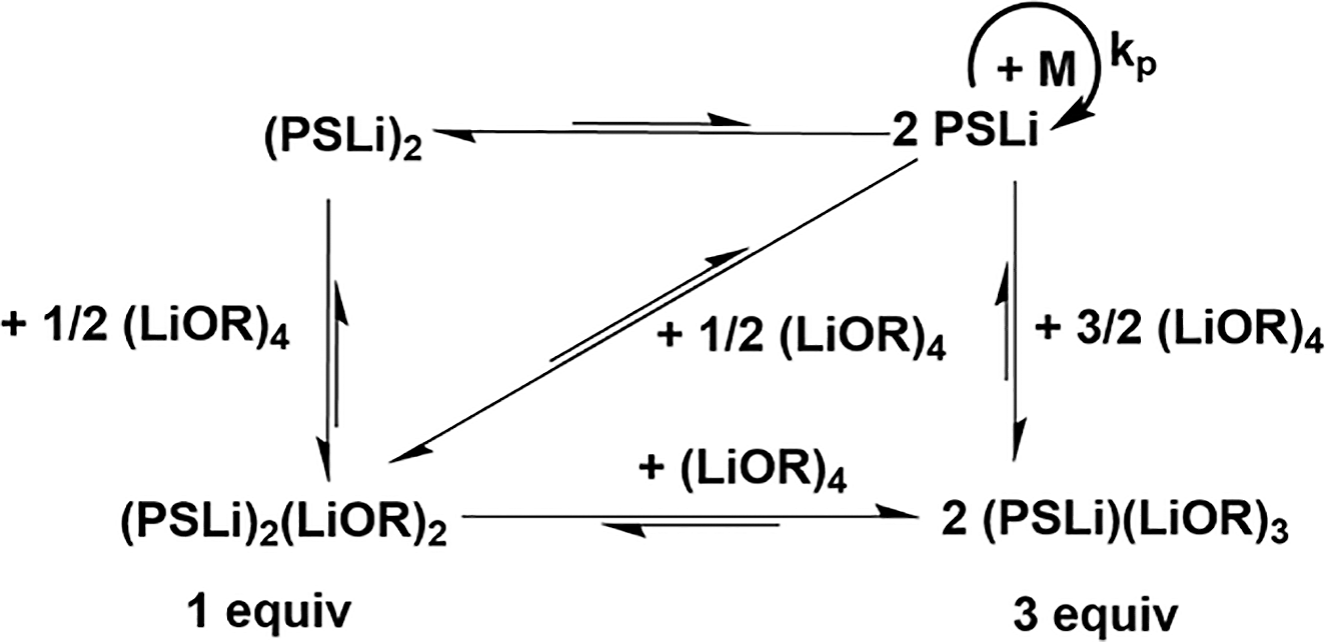
Possible Aggregates of Polystyryllithium in the Presence of Lithium Alkoxides

Figure 1c shows the reactivity ratios vs modifier concentration, calculated from the plots in Figures S15 and S16. With increasing amylate content, the reactivity ratios converge and intersect at an interpolated ratio of ∼5.4 equiv, corresponding to a random copolymerization. At 10 equiv of LiOAm, the gradient is inverted (Table 1 and Figure S21).

Our results for LiOAm quantitatively confirm literature data but significantly deviate from Hsieh’s report on the styrene/butadiene system, who found no change in the comonomer gradient.^32,33^ We tentatively explain these differences by the different diene used (butadiene) and the poorer solubility of LiO*t*Bu in cyclohexane.

### Effect of Sodium *tert*-Amylate (NaOAm)

As can be seen in the kinetic data (Table 1, Figure 2, and Figures S10 and S11), NaOAm has a completely different impact on the copolymerization compared to LiOAm. Already substoichiometric amounts are sufficient to increase the rates of both comonomers (see Figure S14b for half-lives), whereby styrene is more accelerated than isoprene. The calculated reactivity ratios and the resulting gradients are given in Table 1 and Figure S22. The underlying fits are shown in Figures S17 and S18. Increasing the modifier content to 0.75 equiv accelerates the styrene polymerization by two orders of magnitude, while the isoprene rate only increases by a factor of 5 (Figure S14). This leads to a complete inversion of the reactivity ratios and a rather steep inverted gradient (*r*_S_ = 5.11 and *r*_I_ = 0.20). An almost random copolymerization is achieved with only 0.17 equiv of NaOAm, similar to the results published by Hsieh et al. and comparable to the effect of the bidentate ether modifier 2,2-di­(2-tetrahydrofurfuryl)­propane (DTHFP).^24,32^ Thus, sodium alkoxides are much stronger modifiers than the previously investigated THF (random copolymerization at ca. 8 equiv).^17,24^

**Figure 2:**
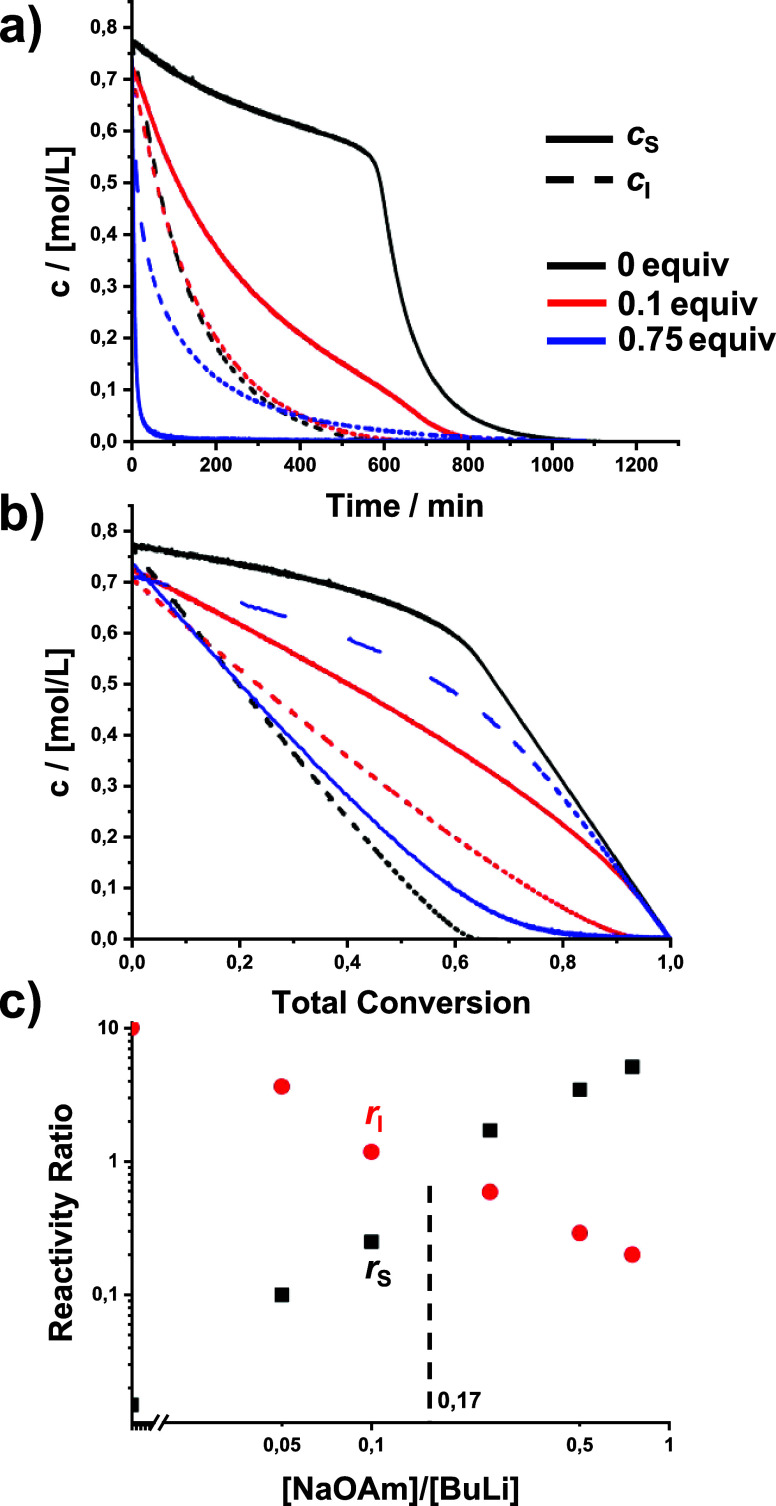
Effect of sodium *tert*-amylate (NaOAm) on the copolymerization kinetics. (a) Time–conversion plots, (b) plots of comonomer concentrations versus total conversion, and (c) reactivity ratios. Red circles: isoprene, black squares: styrene. The dashed line indicates *r*_S_ = *r*_I_ = 1 and thus (ideal) random copolymerization. Please note the double-logarithmic scales.

Our results are consistent with gradients reported by Hsieh and Wofford for the homo- and copolymerizations of styrene and butadiene initiated by NaO*t*Bu/*n*-BuLi, but they differ significantly from the reactivity ratios (*r*_S_ = 0.42, *r*_I_ = 0.3) reported by Arest-Yakubovich et al. for a pure sodium initiator.^31,32,62^ This might indicate that the alkoxide ions play a role in the copolymerization by forming mixed complexes with the PI and PS chain ends (Scheme 3). The mechanism will be discussed further below in comparison with the other modifiers.

### Effect of Potassium *tert*-Amylate (KOAm)

Already minute amounts of KOAm have a dramatic impact on the copolymerization kinetics (Table 1, Figure 3, and Figures S12 and S13). As the potassium content increases, the rate of styrene consumption increases by two orders of magnitude at only 0.25 equiv, while the rate of isoprene consumption and reaction times for full conversion remain constant (Figure 3 a). Hsieh and Wofford found an increase in the rate of butadiene homopolymerization only at more than 0.2 equiv of KOtBu.^31^ In contrast, ethers also affect the polymerization rate of isoprene.^17,26^ The reactivity ratios and gradients are summarized in Table 1, Figure 3c, and Figure S23 (for fits, see Figures S19 and S20). As little as 0.037 equiv of KOAm is necessary to obtain a random copolymerization. A further increase of KOAm concentration leads to complete inversion of the gradient and to a tapered, block-like copolymer. Remarkably, the reactivity ratios (*r*_S_ = 13.8 and *r*_I_ = 0.07) obtained with 0.25 equiv of KOAm are similar to those obtained with 2500 equiv (29 vol-%) of THF. These results are in good agreement with qualitative and semiquantitative results on the S/B copolymerization published by various authors.^32,60,61,64,65,71,79–82^ However, they significantly differ from results published by Nakhmanovich et al., who investigated a pure organopotassium initiator (*r*_S_ = 3.3; *r*_B_ = 0.12), indicating that both counterions and the alkoxide affect the polymerization mechanism. A comprehensive discussion will be given in a later section of this paper.

**Figure 3:**
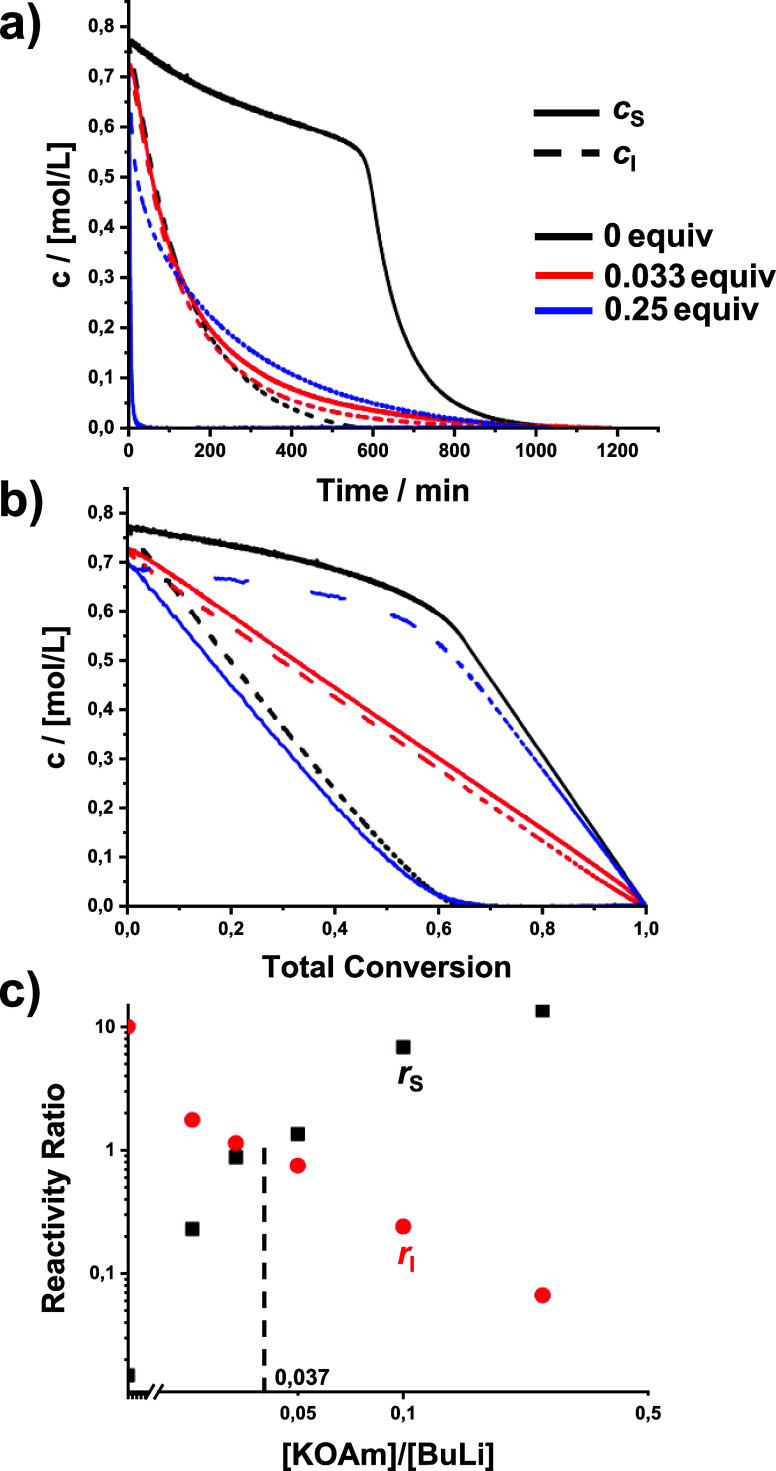
Effect of potassium *tert*-amylate (KOAm) on the copolymerization kinetics. (a) Time–conversion plots, (b) plots of comonomer concentrations versus total conversion, and (c) reactivity ratios vs concentration. Red circles: isoprene, black squares: styrene. The dashed line indicates *r*_S_ = *r*_I_ = 1 and thus (ideal) random copolymerization. Please note the double-logarithmic scales.

### Determination of Blockiness

The so-called “blockiness” is defined as the fraction of two or more consecutive styrene units in the copolymer, as analyzed by a characteristic shift of the *ortho* protons in ^1^H NMR.^83,84^ The stacked NMR spectra are shown in Figures S24–26. The resulting blockiness values as a function of modifier equivalents are given in Table 1 and Figure 4. For details regarding determination of the blockiness, see the Supporting Information, Section 4.2.^8,17,24^ The general trend for Lewis base modifiers is that with converging reactivity ratios, the blockiness decreases, until a minimum for random copolymerization is reached.^8,17,24^ Steube et al. observed this for THF as a modifier. The blockiness decreased from 75% in cyclohexane to 13% for random copolymerization with 4 to 8 equiv THF (Figure S27).^17^

For LiOAm and NaOAm, we observe a decrease to only 50%, indicating a deviation from an ideal randomness. KOAm shows an even more unexpected behavior: the blockiness steadily increases up to 92% at complete gradient inversion. It is remarkable that at random copolymerization (0.037 equiv), we observe a higher blockiness than in a tapered, block-like copolymer obtained in pure cyclohexane. This indicates the formation of short PS blocks. We explain this with an altered polymerization mechanism, which will be discussed in detail in a later section. To confirm this assumption, a tapered copolymer synthesized in pure cyclohexane and a random one synthesized using 0.033 equiv of KOAm were submitted to oxidative degradation (for details, see Supporting Information, sections 1.4 and 4.2).^85,86^ SEC distributions of the polymers before and after degradation are shown in Figure S28. NMR (Figure S29) confirmed that all double bonds were successfully degraded. As expected, degradation of the tapered copolymer yielded a pure PS block with a molecular weight of 36 kg/mol and low dispersity, whereas the random copolymer was broken down into smaller PS blocks with a molecular weight of ca. 1700 g/mol and a dispersity of 2.3.

**Figure 4:**
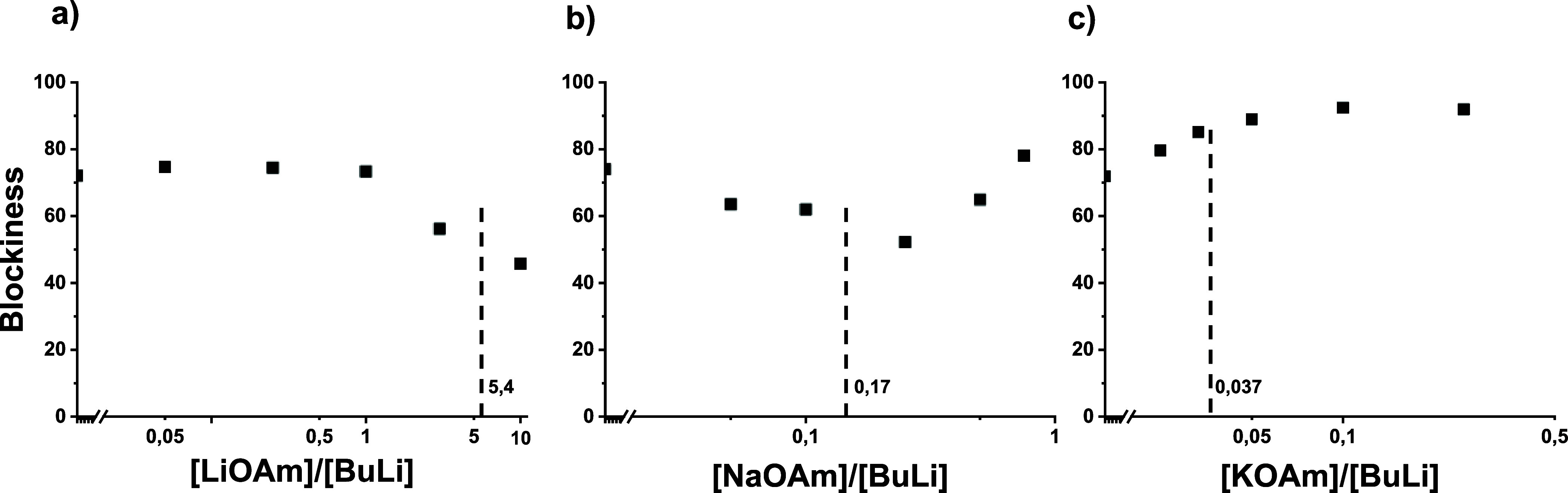
Blockiness of P­(S-*co*-I) synthesized as a function of the MOAm/BuLi ratio. The vertical line represents random copolymerization.

### Microstructure of Isoprene Units

The microstructure of diene polymers is a key feature for all application areas. ^1^H NMR spectroscopy was used to determine the microstructure of the isoprene units. Exemplary ^1^H-, ^13^C-, COSY, HSQC, and HMBC spectra of copolymers synthesized in the presence of 0 eq, 0.75 equiv NaOAm, and 0.25 equiv KOAm are shown in Figures S30–S44, and the results are given in Table 1 and Figure 5. The microstructure of the copolymers synthesized in pure cyclohexane consists of 94% 1,4- and 6% 3,4-units, which is in good agreement with literature.^8,16,17,23,30,87^ It is common that with increasing amount of modifier, the 1,4-content decreases and the 3,4- and 1,2-contents increase. A similar behavior has been reported for ether- or amine-based modifiers. As shown in Figure 5, the extent to which this microstructure changes varies greatly for the various counterions and is dramatically different for KOAm.

**Figure 5:**
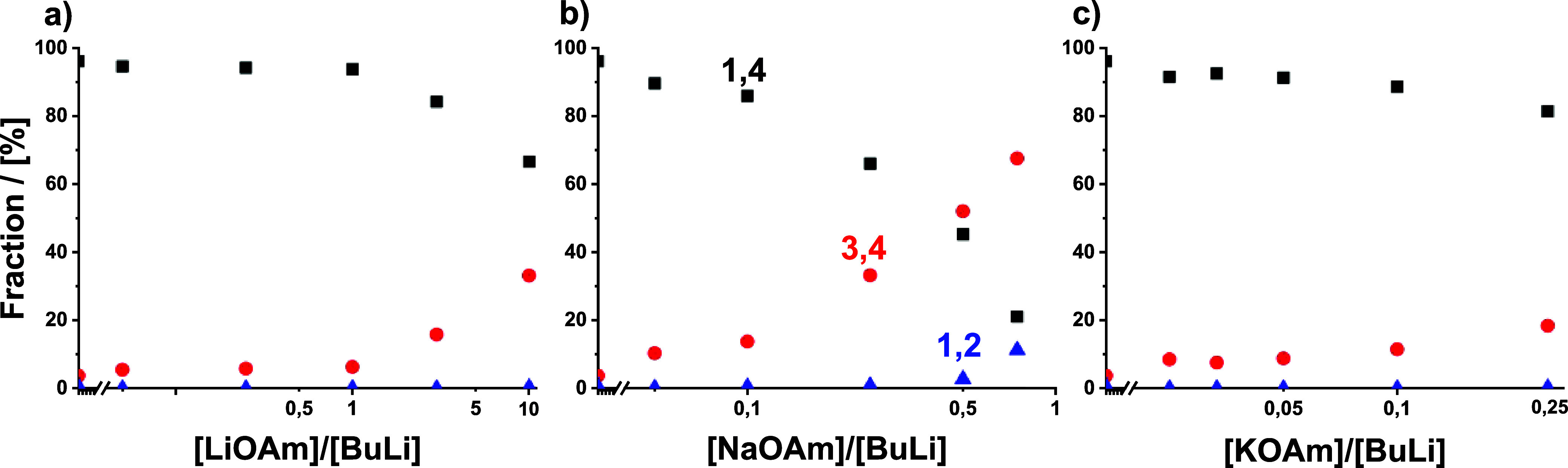
Microstructure of the isoprene units (1,4: black squares; 3,4: red dots; 1,2: blue triangle) obtained in the presence of *tert*-amylates.

LiOAm affects the microstructure only in overstoichiometric concentrations, raising the vinyl content from 6 to 33%. This is similar to the results of Makowski et al.^48^ for polybutadiene at slightly lower alkoxide concentrations but contradicts the results of Hsieh,^33^ where LiO*t*Bu had little effect on the polybutadiene microstructure. As discussed above, we suspect that LiO*t*Bu was probably not completely dissolved in Hsieh’s experiments.

Even small amounts of NaOAm have a significant effect on the microstructure, which is in good agreement with literature.^31,54^ Already, 0.75 equiv NaOAm is sufficient to alter the vinyl content from 6 to 79%, similar to the effect of 2500 equiv of THF.^17^ Metallic sodium or organosodium initiators lead to similar microstructures.^36,49,51,52^ Thus, the microstructure of the isoprene units is dominated by the sodium counterion. Overall, sodium ions have the highest impact on the microstructure and are used to synthesize polymers with high vinyl unsaturation content.^32,49,50,88^

Surprisingly, KOAm, the most efficient modifier in this study, changed the microstructure only to a small extent (8 and 18% vinyl units at random and at full inversion, respectively). This renders KOAm highly interesting for the synthesis of S/I or S/B random copolymers with a high 1,4 microstructure, as documented by a number of patents.^2,3,69,70^ These results are in contradiction to previous results for homopolybutadiene, where a higher vinyl unsaturation of up to 50% was achieved by using potassium alkoxides in sub- and overstoichiometric concentrations.^31,41,47,54^ However, it is in good agreement with the results published by Kirchevskaya et al. for polyisoprene.^47^ We explain the differences between butadiene and isoprene units with the additional methyl group, increasing sterics, and a change in the microstructure determination method from infrared to ^1^H NMR spectroscopy, which is more accurate.^23,30,89^ In pronounced contrast, potassium metal and alkylpotassium initiators led to vinyl contents of about 50%.^29,49,52,53,90,91^ This is a strong indication that both lithium, potassium, and the alkoxide affect isoprene polymerization.

### Comprehensive Discussion

Our kinetic and microstructural investigation has shown strong differences in the behavior of the three modifiers. Retardation of the copolymerization by more than one equiv of LiOAm was explained by the existence of various mixed aggregates and a decrease of free, nonaggregated PS-Li and PI-Li. However, the only partial decrease of the blockiness and the increased vinyl content of the isoprene units seem to indicate that a part of the active species are mixed complexes, e.g., (P–Li)­(LiOAm)_3_; see Scheme 3.

With sodium and potassium amylate, we assume complete metal exchange (Scheme 2), but since they are used in deficit, only a fraction of polymer chains can be coordinated with sodium or potassium. This fraction depends on the nature of the chain end and the alkali metal (Scheme 4). The chain ends with Na^+^ or K^+^ counterions are more reactive than the lithiated ones (*k*_p,Mt_ > *k*_p,Li_), explaining the observed increase in the polymerization rate. The species formed in the metal exchange reaction can form mixed aggregates with the residual Li, Na, or K alkoxides, similar to the case in Scheme 3 or a two-state equilibrium (Scheme 4). The open question is whether these mixed complexes participate in the polymerization.

**Scheme 4:**
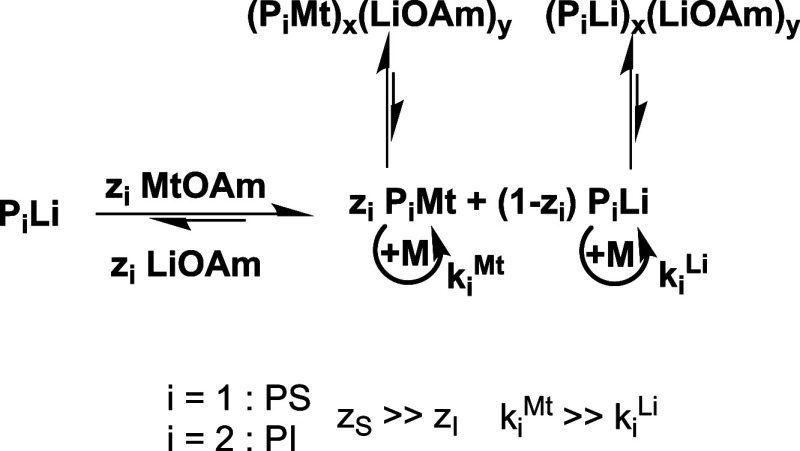
Effect of NaOAm and KOAm on the S/I Copolymerization

This question is difficult to answer for the NaOAm modifier. On the one hand, microstructures observed in our system are similar to those obtained with metallic sodium and organosodium initiators, indicating that the mechanism is dominated by a metal exchange in the two-state equilibrium.^36,49,51^ On the other hand, the reactivity ratios in our system significantly differ from those published for pure organosodium initiators, indicating participation of a mixed complex (PMt)_*x*_(LiOAm)_*y*_.^62^ Furthermore, Arest-Yakubovich reported that addition of lithium alkoxide to 2-ethylhexylsodium suppresses chain transfer to toluene.^51^ Thus, we conclude that both counterions and the alkoxide are relevant for the polymerization.^36,37,44,51,62,63^

KOAm has the strongest effect on the kinetics but still polymerizes isoprene in the predominant 1,4-microstructure. To the best of our knowledge, this is the only known modifier capable of altering reactivity ratios by accelerating styrene polymerization and, nevertheless, having a negligible effect on isoprene polymerization and its microstructure. We explain this by the two-state polymerization mechanism in Schemes 2 and 4, where the equilibrium remains left for isoprene but shifts right for styrene.^31,32,37,45,73^ According to the HSAB (hard and soft acids and bases) concept,^92^ the delocalized (soft) polystyryl anions predominantly coordinate with soft (potassium) cations, whereas the (hard) polydienyl anions prefer the hard lithium cations, explaining the predominant 1,4-units.^45,69,70^ Thus, styrene polymerization is strongly accelerated, leading to the observed effect of the reactivity ratios. At the same time, the microstructure of the isoprene units remains unaltered (Table 1 and Figure 5) and blockiness increases (Figure 4).

The unexpected and unprecedented increase of blockiness and the formation of short PS blocks, even despite random copolymerization at ca. 1/30 equiv of KOAm, need special consideration. We explain this effect with a two-state polymerization mechanism. At 1/30 equiv of KOAm, there can be a potassium counterion at one of the 30 polymer chain ends, most probably a PS chain end. Since they are considerably more reactive, the PS-K chain ends polymerize as much styrene as the other 29 PS-Li and PI-Li chain ends preferably polymerize isoprene, since *k*_SI_ ≫ *k*_SS_.^17^ Based on rapid exchange of the counterions, monomodal polymers are accessible that consist of many short styrene and isoprene blocks, which increases the overall blockiness, since only two to three styrene units are required for the characteristic NMR shift of the *ortho* protons.^84^ This is in contrast to previously studied Lewis bases, e.g., ethers, which lead to fully random copolymers.^8,17,23,24^ Therefore, potassium alkoxide as a modifier enables the decoupling of the diene microstructure from the reaction kinetics.

### Thermal Properties

The glass transition temperatures, *T*_g_, of the P­(S-*co*-I) copolymers were investigated by DSC. The results are presented in Table 1 and Figure 6. The second heating cycles are shown in Figures S45–S47.

**Figure 6:**
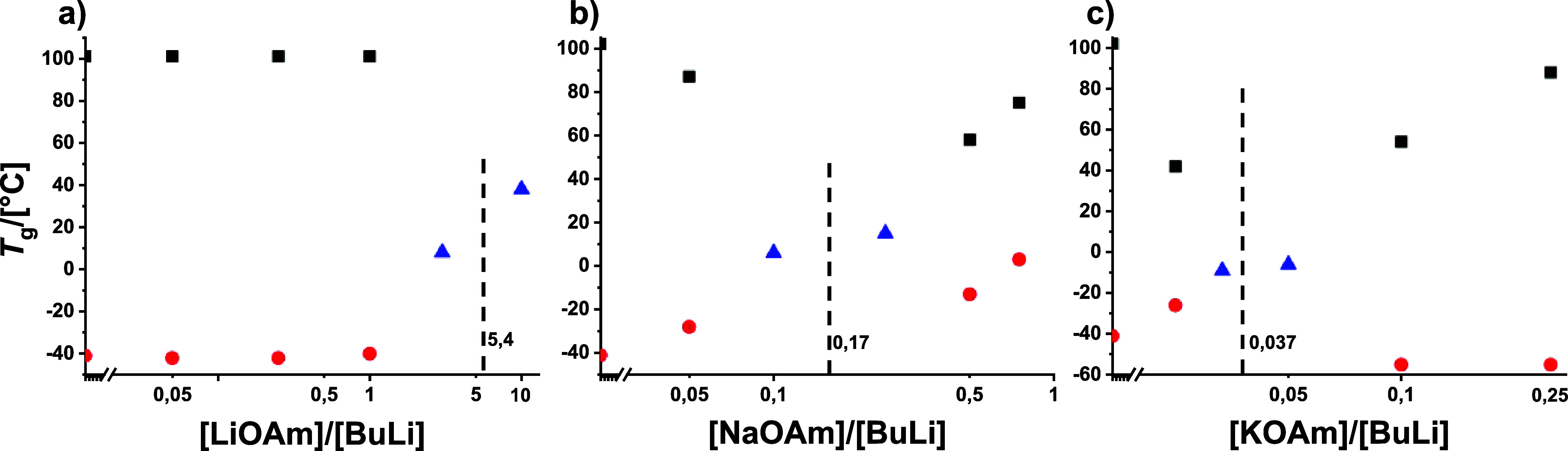
Glass transition temperatures of P­(S-*co*-I) obtained in the presence of *tert*-amylates. Black squares: PS-rich phase, red dots: PI-rich phase, blue triangles: mixed phase. The vertical lines indicate random copolymerization.

As expected, the addition of up to 1 equiv of LiOAm had no effect on the glass transition temperature of the resulting phase-separated polymers. The values for the PS-rich phase at 100 °C and for the PI-rich phase at about −40 °C are in good agreement with literature.^17^ The *T*_g_ of the PI-rich phase is almost 20 °C higher than that of the 1,4-PI homopolymer,^93,94^ because of its contamination by short styrene units. Increasing the modifier content to ≥3 equiv of LiOAm shifts the two distinct glass transition temperatures into one mixed phase. This is explained by the change in reactivity ratios (Table 1 and Figure 1c) and the increasing vinyl unsaturation of the diene.^55^

The glass transition temperature of copolymers polymerized with NaOAm behaves as expected from ether-based modifiers.^8,17,23,24^ At 0.05 and ≥0.5 equiv of NaOAm, two distinct glass transition temperatures are observed, indicating phase separation due to the pronounced tapered structure. For 0.1 and 0.25 equiv of NaOAm, we again find phase mixing, as expected from the reactivity ratios (Table 1 and Figure 2c). When the phases are separated, the *T*_g_ of the PS-rich phase is below the *T*_g_ of the PS homopolymer, since the polymer segments exhibit isoprene defects, and in a similar manner, the *T*_g_ of the PI-rich phase increases due to the microstructure change (Figure 5).

The glass transition temperatures of copolymers polymerized with up to 0.05 equiv of KOAm show a behavior similar to that with the other amylates. In particular, the copolymers formed at 0.033 and 0.05 equiv show a mixed *T*_g_, indicating the absence of phase separation in spite of the formation of short blocks. These blocks are too short to undergo phase separation. At ≥0.1 equiv KOAm, the *T*_g_ of the PI-rich phase drops to −55 °C, a value that is 14 degrees lower than that of a copolymer synthesized in pure cyclohexane. We explain this previously unreported phenomenon by the polymer composition. This tapered copolymer consists of a PS block contaminated with isoprene units (*T*_g_ lowered), predominantly grown with K^+^ counterions. After the tapered region, a pure PI block exists, with a high (88 and 81%) 1,4-content due to the preferred isoprene polymerization with lithium as a counterion. Since the 1,4-content in pure cyclohexane is still higher (95%), but the PI-rich phase has a lower *T*_g_, we assume that the styrene contaminations have a stronger impact on *T*_g_ than the increased content of vinyl units. A tapered copolymer with a pure PI block can also be obtained in the presence of 2500 equiv of THF, which decreases the 1,4-content to 25% and thus increases the *T*_g_ to 5 °C.^17^

## Conclusions

We have presented the first in-line NIR kinetic investigation of the copolymerization of styrene and isoprene initiated by *sec*-butyllithium (BuLi) in cyclohexane in the presence of alkali metal alkoxides as modifiers to calculate reactivity ratios and comonomer gradients. Additional information on the underlying mechanisms was obtained from NMR spectroscopy, namely, the blockiness and the microstructure of the diene units. The glass transition temperatures of the gradient copolymers correlate with the copolymer microstructure. All investigated amylates affect the rate of polymerization, the reactivity ratios, the comonomer gradient, and the diene microstructure, but at very different concentrations and with different reaction mechanisms. The general trend in modifier strength is Li < Na < K. The results show that these Lewis acid (μ-type) ligands act completely different to the well-known Lewis base (σ-type) ligands, e.g., ethers or amines.

LiOAm at overstoichiometric concentrations decreases the rate of polymerization of both monomers to different degrees, affecting the respective reactivity ratios. This is due to a decrease in the concentration of active chains by the formation of mixed aggregates. The only partial decrease in the blockiness and the moderately increased vinyl content of the isoprene units suggest that a part of the active species are mixed complexes.

Substoichiometric addition of NaOAm accelerates styrene propagation but has only a minor effect on the rate of isoprene conversion. However, it significantly changes the microstructure of the diene units, similar to that of metallic or organosodium initiators. Thus, exchange of the lithium counterion to sodium leads to the predominantly active species of both PS and PI chain ends (Scheme 4). The reported fact that the addition of LiO*t*Bu to an organosodium initiator strongly decreases chain transfer to toluene indicates a contribution of mixed complexes. Thus, sodium alkoxides are promising modifiers for the synthesis of high vinyl polydienes suitable for postpolymerization modification.

KOAm selectively accelerates styrene polymerization even at minute concentrations, whereas the isoprene rate and microstructure are unaffected. An unexpected phenomenon is the formation of short blocks in a random copolymer. This is explained by the two-state polymerization mechanism involving a selective exchange from polystyryllithium to potassium, resulting in a very low *T*_g_ of −55 °C of the isoprene units. This unique feature of decoupling the reaction kinetics from the diene microstructure is the most impressive capability of potassium *tert*-amylate, rendering it a perfect choice for the synthesis of random copolymers and thermoplastic elastomers with a high content of 1,4-isoprene units.

## Supplementary Material



## Notes

Comprehensive compilation of additional data including detailed experimental description, plots of each kinetic experiment, molar and volume compositional gradients, and NMR, SEC, and DSC results of each polymer (PDF)
