# Supplementary material for: Dramatic Effect of Alkali Metal Alkoxides on the Anionic Copolymerization of Styrene and Isoprene
Source: Macromolecules. 2025 Jun 16;58(13):6854–64. doi: 10.1021/acs.macromol.5c00975 (PMC12257597; doi:10.1021/acs.macromol.5c00975)
Supplement: Supplementary file 1 [file ma5c00975_si_001.pdf]

Supporting Information

Dramatic Effect of Alkali Metal Alkoxides on the Anionic Copolymerization of Styrene and Isoprene

Dominik A. H. Fuchs[1], Holger Frey[1*], Axel H. E. Müller[1*]

[*]Corresponding authors

[1] Johannes Gutenberg-University, Duesbergweg 10-14, 55128 Mainz, Germany
hfrey@uni-mainz.de; muellax@uni-mainz.de

Table of contents

1. Materials and experimental procedure: 2
1.1 Materials: 2
1.2 Instrumentation 2
1.3 Copolymerisation 2
2. Copolymerization kinetics 4
2.1 NIR spectra 4
2.2 Temperature profiles 5
2.3 SEC traces of copolymers 7
2.4 Kinetic plots 8
3. Determination of reactivity ratios: 11
4. Copolymer composition 15
4.1 Molar and volume composition profiles 15
4.2 Blockiness 16
5. Microstrcuture of PI units 20
6. Glass transition temperatures 26
7. References 27

1. Materials and experimental procedure:

1.1 Materials:

All chemicals were purchased from following suppliers: Sigma-Aldrich, Thermo Fisher Scientific Inc, Acros, VWR international and Deutero GmbH. The solution of potassium *tert.* Amylate was kindly provided by Ineos group limited. As inert gas was argon 5.0 used, which was further purified by passing it through three gas wash bottles containing *sec*. BuLi, diphenylethylene and cyclohexane.

1.2 Instrumentation

A description of the near infrared (NIR) set up and data evaluation can be found in previous publications.[1,2] NMR spectra were measured using a Bruker Avance III 600 spectrometer at 600 MHz for ^{1}H- and 151 MHz for ^{13}C-NMR. GPC samples were analyzed on an Agilent 1260 Infinity II set up with MZ-Gel SDPlus plus 10^5/10^3/100 Å columns from MZ-Analysetechnik, Mainz, Germany. All samples were measured at a concentration of 1 mg/ml with toluene as an internal standard with a PS calibration. Thermal DSC analysis of the copolymers were performed with a TA instruments DSC 250 connected to a TA instrument RCS 90 refrigeration system from Waters Corporation. Experiments were performed at a heating and cooling rate of 10K/min with a two-point calibration of indium and *n*-ocatane.

1.3 Copolymerisation

1.3.1 Purification of materials

The amylates used were not further dried, but their water content was quantified *via* Karl-Fischer titration to below 60 ppm of water and therefore no further drying was considered necessary.

Every glass flask used was flame dried three times under high vacuum (10^{-3} mbar) before use and all reactions and drying processes were carried out under schlenk conditions. A pressure valve maintained a constant overpressure of 50 mbar argon to ensure an inert gas atmosphere. The monomers were filtered through basic alumina to remove stabilizers and then dried over CaH_2 for a day. After degassing through three freeze thaw cycles, the monomers were cryotransferred to $Al(Oct)_3$ and further dried for a day. After the drying process, the monomers were cryotransferred into a graduated ampoule, with less than 2 $\%_{Vol}$ loss in relation to the total volume of the monomers. The solvent cyclohexane was dried under reflux with sodium and benzophenone. After the blue color appeared, the solvent was transferred into the Morton flask glass reactor.

1.3.2 Copolymerization in the presence of different amylates

All polymerizations were carried out under the same conditions, only the amount of modifier was varied. The reaction flask was equipped with the NIR probe, a temperature probe and an additional glass joint. To maintain a constant reaction temperature, the flask was placed into a water bath that was cooled via a cryostat. In all reactions, the reaction heat was not exceeded by more than 10 °C (Figures S2-S4), and previous publications have shown that the effect of this temperature increase can be neglected as compared to the modifier effects.[1,3] Since mixtures of MtOR and BuLi are known as Lochmann-Schlosser superbases,[4,5] which have strong deprotonating abilities, due to their metal exchange (see Figure 2). These are no longer soluble in

hydrocarbon solvents and form possible side reactions (Scheme S1).[6] Carlotti *et al.* could prove that it is essential for a controlled copolymerization that the initiator system of MtOR/BuLi had no preformation time to avoid side reactions and that MtOR had to be used in deficit to avoid crosslinking.[5,7–10] Therefore we have the goal of using the alkoxide in deficit and giving the initiator no preformation time.

The synthesis of P(S-*co*-I) in cyclohexane with 0.25 equiv. of [KOAm] is described here as example. First, 600 ml cyclohexane was added into the reaction flask followed by the modifier [KOAm] (0.25 equiv., 0.79 ml of a 0.33 mol/L solution in cyclohexane) to measure a background NIR spectra using Omnic software. An equimolar mixture of the monomers styrene (55.3 ml, 0.483 mol) and isoprene (48.35 ml, 0.483 mol) was added, and the polymerization was initiated by the injection of 0.8 ml (0.26 mmol, 1eq) of a 1.3 mol/L solution of *sec.* BuLi in cyclohexane/hexane (92:8). After initiation, the colorless solution turned dark red, indicating the preferential polymerization of styrene. After 1000 minutes the polymerization was complete and was terminated by adding 2 ml of degassed isopropanol in argon counterflow. The synthesized polymer was precipitated in a 1:3 mixture of isopropanol:methanol. The resulting colorless polymer was dried under reduced pressure and stored at -20°C.

For the kinetic investigation 13,000 to 20,000 NIR-spectra were recorded in the wavelength range from 5900 to 6250 cm^{-1} and analyzed by deconvolution, in analogy to our previous publications.[1–3,11,12] The calibration spectra of isoprene, styrene, polystyrene and polyisoprene are shown in Figure S1. The polyisoprene spectrum was calculated by subtracting the polystyrene spectrum from the spectrum at full conversion and normalizing it by the isoprene concentration, determined by the deconvolution of the starting spectrum. This is necessary because the NIR spectrum of PI is dependent on the microstructure, which depends on the modifier used.

1.4 Oxidative degradation

The oxidative degradation is performed analog to Corbin et al., with the exception that the polymer is not precipitated, but analyzed from the crude solution.[13]

To a solution of 1.5 g polymer in 200 mL benzene is 4.0 g *meta*-chloroperoxybenzoic acid (mCPBA) added and the solution stirred for 15 hours at room temperature. After removing the solvent under reduced pressure, the residue was dissolved in 150 mL dioxane and a solution of periodic acid (3.5 g in 20 mL water) is added. The solution was stirred for 24 hours at room temperature and then heated to 65°C for 30 minutes and the solvents removed under reduced pressure. The dried residue was washed with diluted NaOH and water. The obtained residual was analyzed via SEC without prior work up. Please note that the PS oligomers carry aldehyde endgroups, which can affect the polystyrene calibration.

Scheme S1: Hypothetic mechanism of the elimination of potassium hydride.[6]

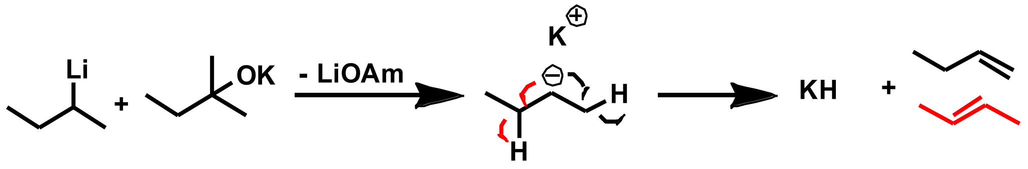

2. Copolymerization kinetics

2.1 NIR spectra

The measured (S, I and PS) and calculated (PI_{CyH} and $PI_{0.75eq\ Sodium}$) are displayed here. The microstructure depended PI spectrum is calculated by subtracting the PS spectrum from the final spectrum. These spectra are then used to perform a deconvolution to calculate the concentration of each component for each time point.

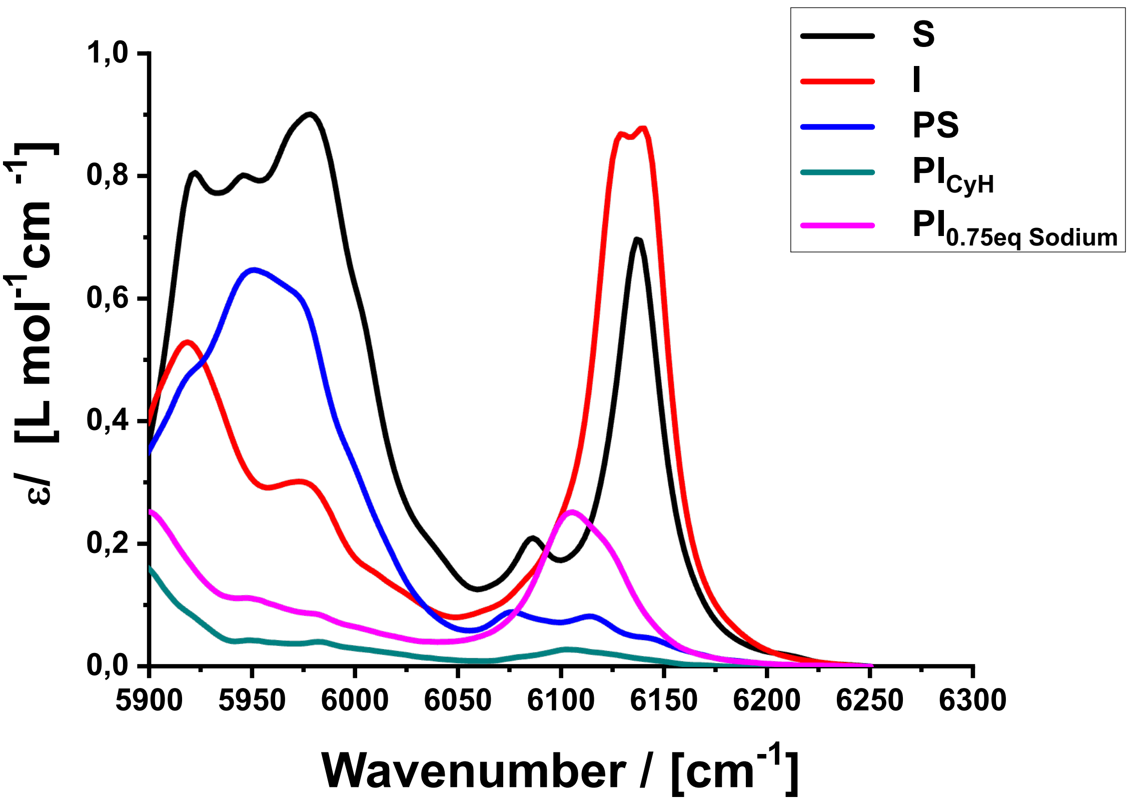

Figure S1: Measured molar attenuation coefficients of all in the reaction present reagents (S, I) and products (PS, PI). PI_{CyH} represents the spectra of polyisoprene synthesized in cyclohexane with the typical microstructure of (94% 1,4-; 6% 3,4-) and $PI_{0.75\ equiv\ NaOAm}$ the spectra of polyisoprene synthesized in cyclohexane with 0.75 equiv of NaOAm and a microstructure of (21% 1,4-; 68% 3,4-; and 11% 1,2-;) as determined by NMR spectroscopy.

2.2 Temperature profiles

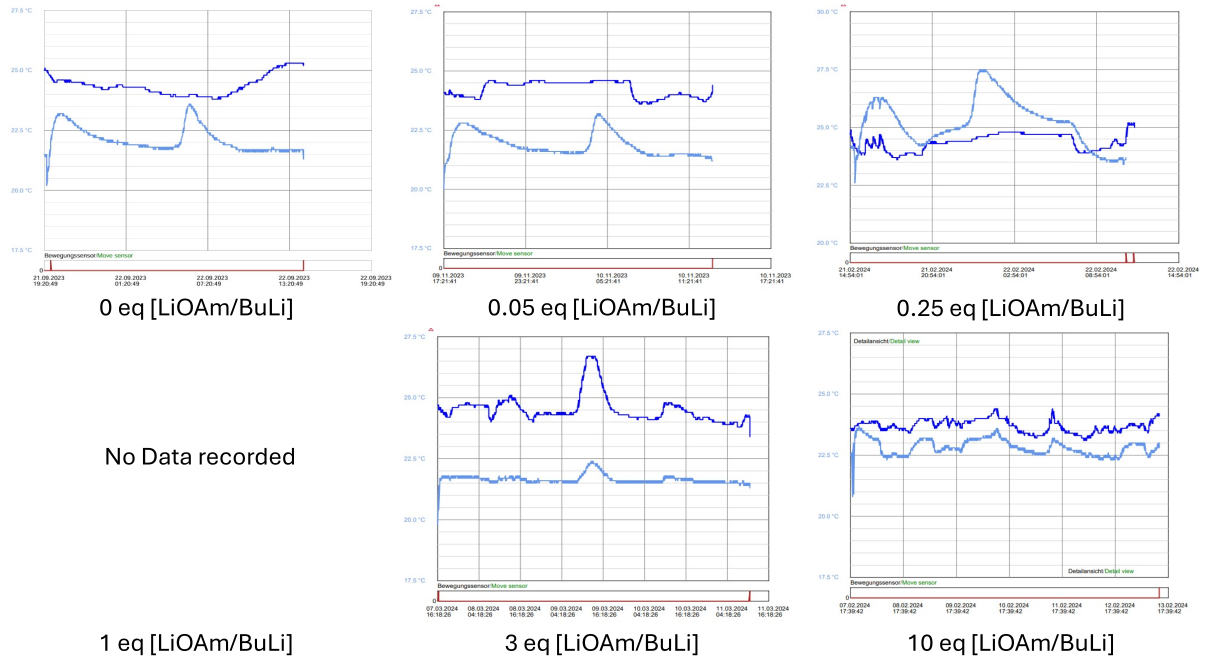

Figure S2: Temperature recordings inside the reaction vessel for various [LiOAm/BuLi] ratios (light blue: temperature in the reaction vessel, drak blue room temperature).

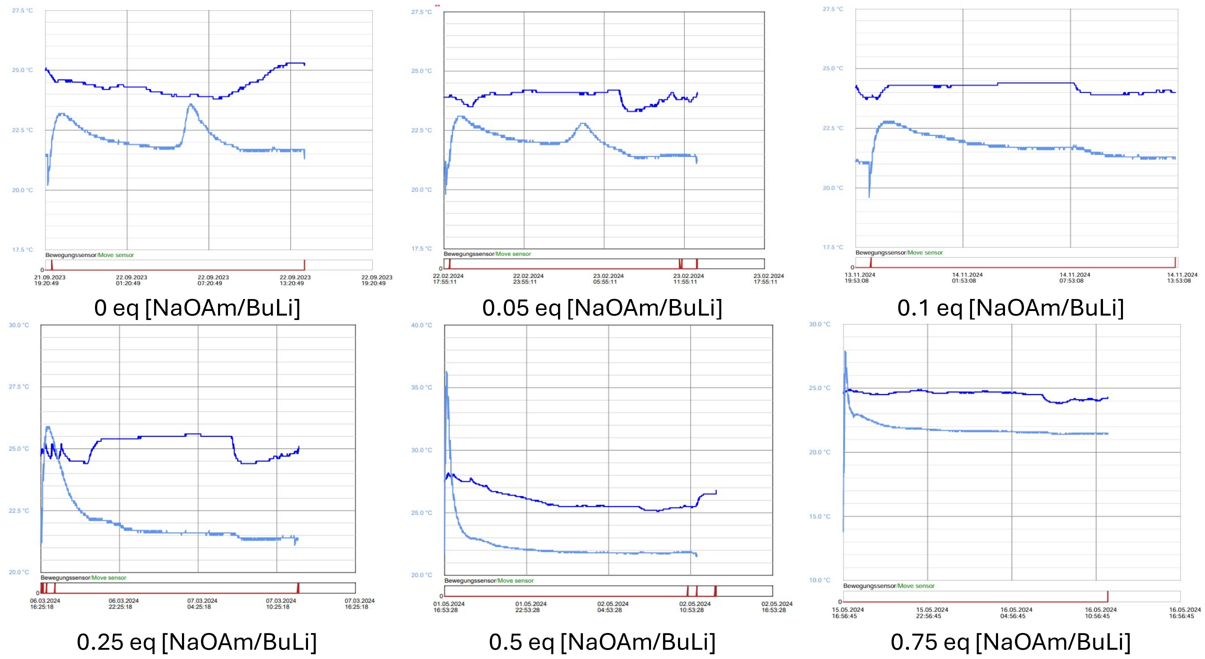

Figure S3: Temperature recordings inside the reaction vessel for various [NaOAm/BuLi] ratios (light blue: temperature in the reaction vessel, drak blue room temperature).

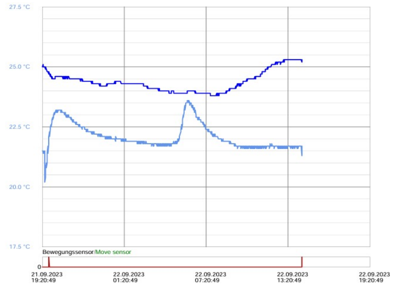

0 eq [KOAm/BuLi]

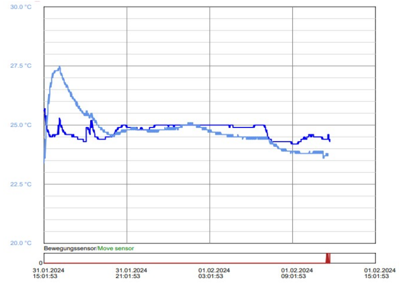

0.025 eq [KOAm/BuLi]

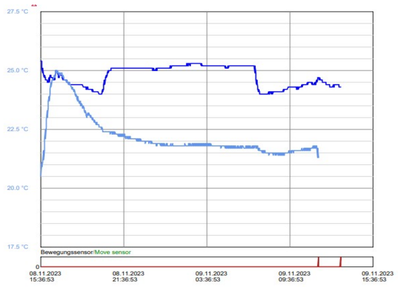

0.033 eq [KOAm/BuLi]

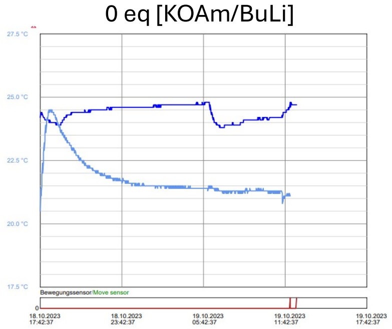

0.05 eq [KOAm/BuLi]

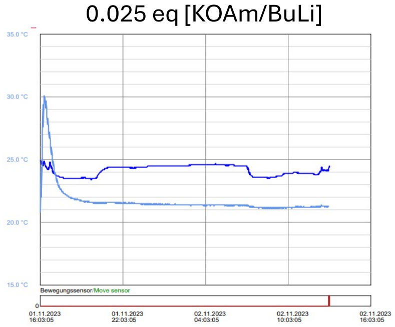

0.1 eq [KOAm/BuLi]

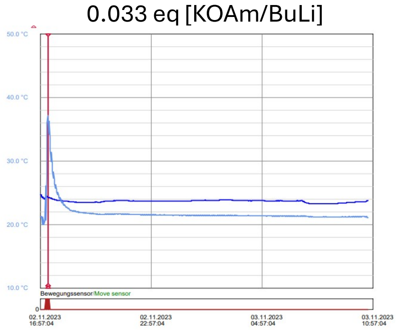

0.25 eq [KOAm/BuLi]

Figure S4: Temperature recordings inside the reaction vessel for various [KOAm/BuLi] ratios (light blue: temperature in the reaction vessel, drak blue room temperature).

2.3 SEC traces of copolymers

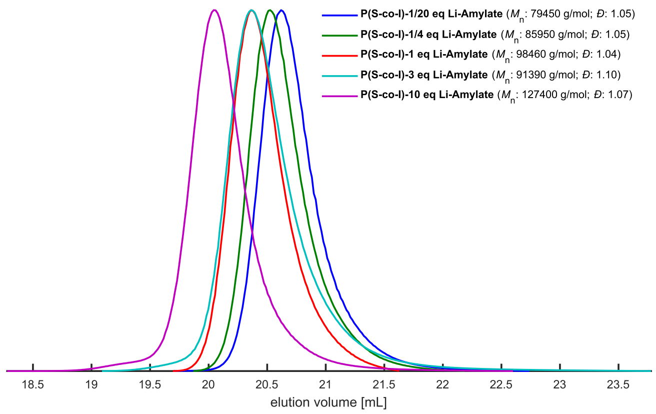

Figure S5: SEC (THF) traces of P(S-*co*-I) with different [LiOAm/BuLi] ratios and PS calibration.

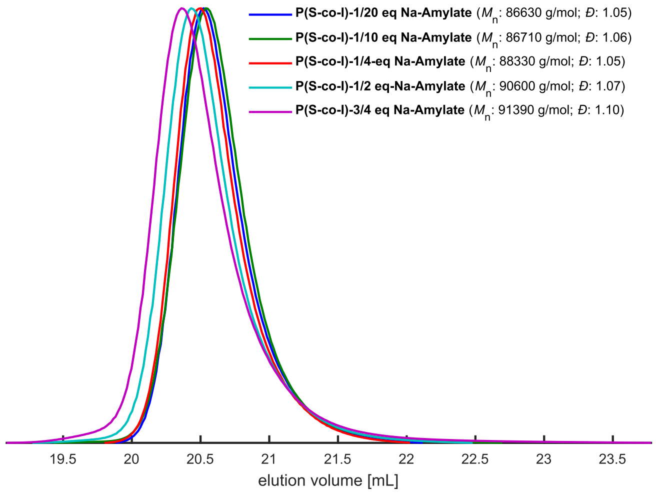

Figure S6: SEC (THF) traces of P(S-*co*-I) with different [NaOAm/BuLi] ratios and PS calibration.

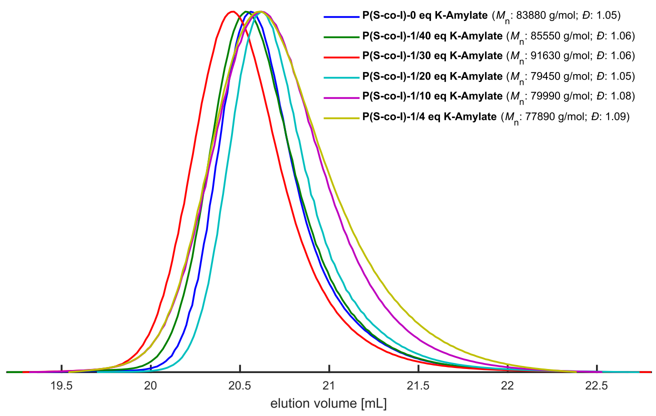

Figure S7: SEC (THF) traces of P(S-*co*-I) with different [KOAm/BuLi] ratios and PS calibration.

2.4 Kinetic plots

2.4.1 LiOAm

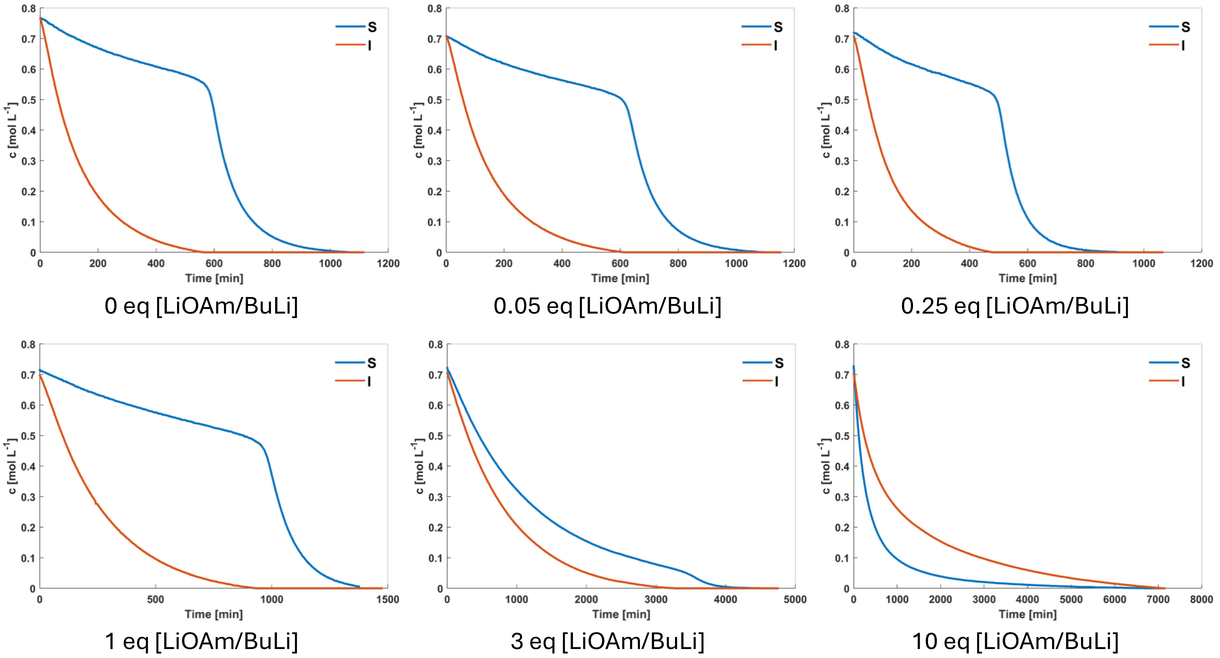

Figure S8: Individual time-conversion plots at various [LiOAm/BuLi] ratios.

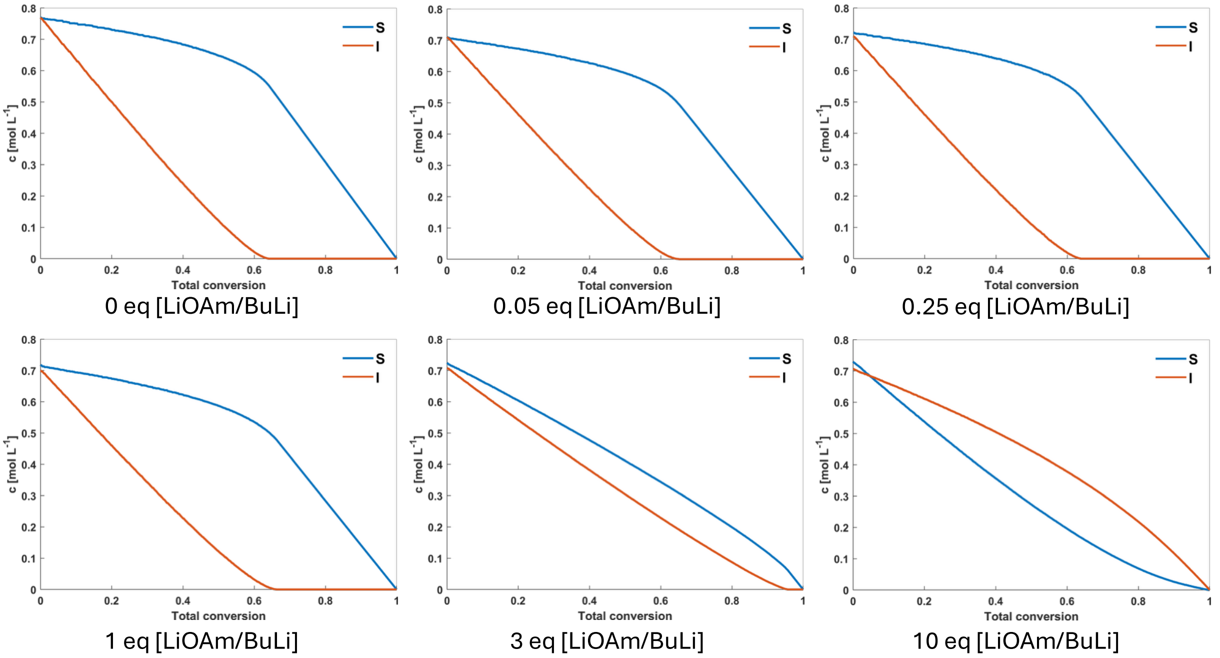

Figure S9: Individual monomer concentrations as a function of total conversion for various [LiOAm/BuLi] ratios.

2.4.2 NaOAm

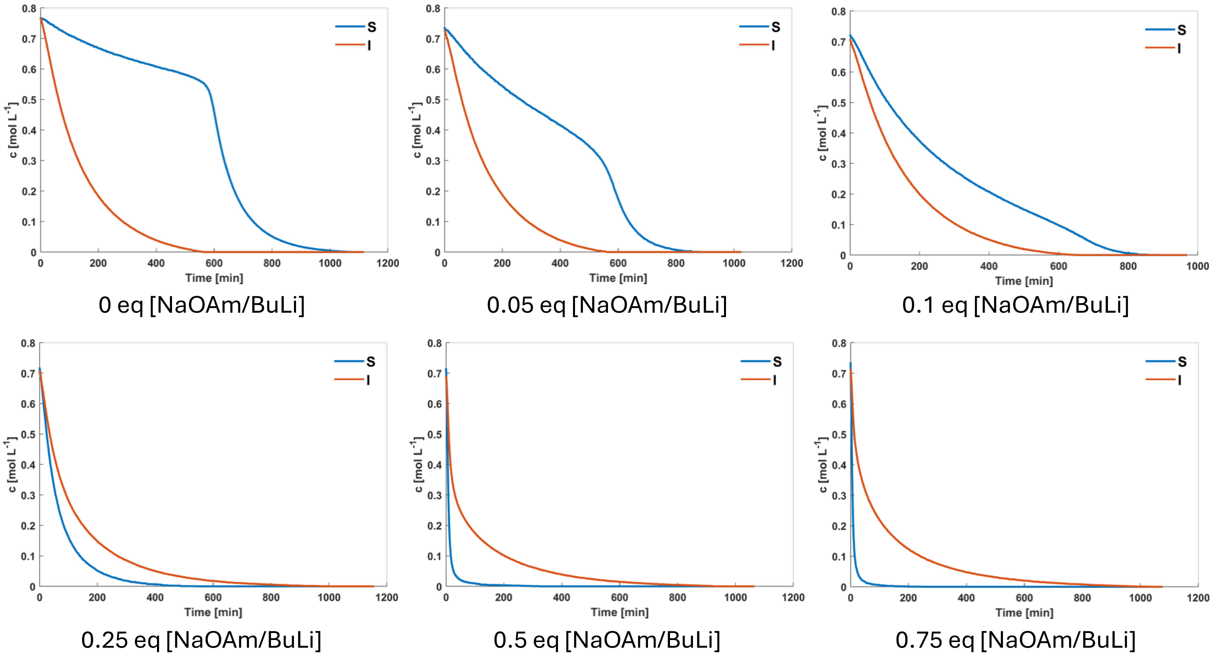

Figure S10: Individual time-conversion plots at various [NaOAm/BuLi] ratios.

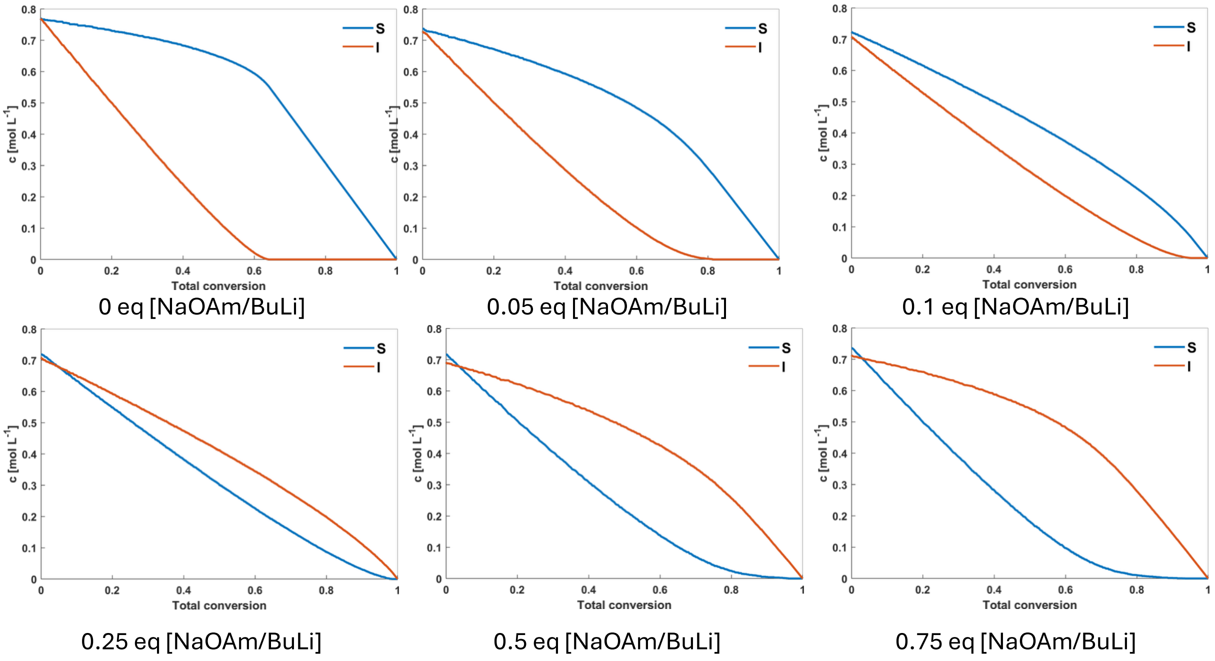

Figure S11: Individual monomer concentrations as a function of total conversion for various [NaOAm/BuLi] ratios.

2.4.3 KOAm

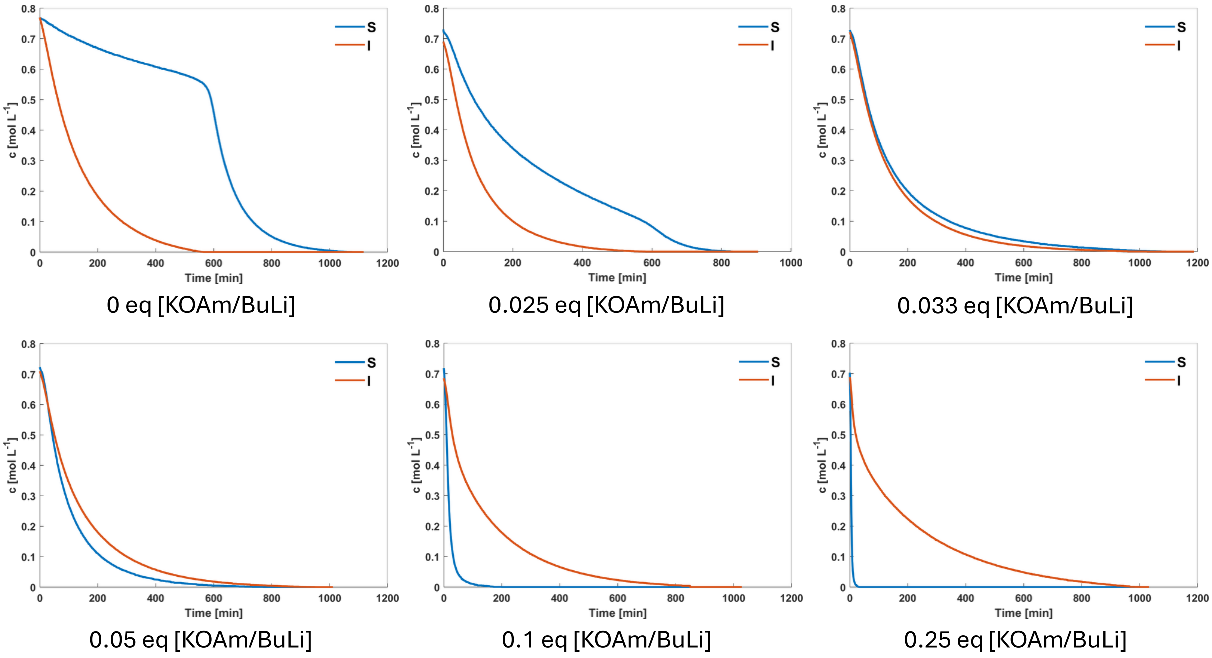

Figure S12: Individual time-conversion plots at various [KOAm]/BuLi] ratios.

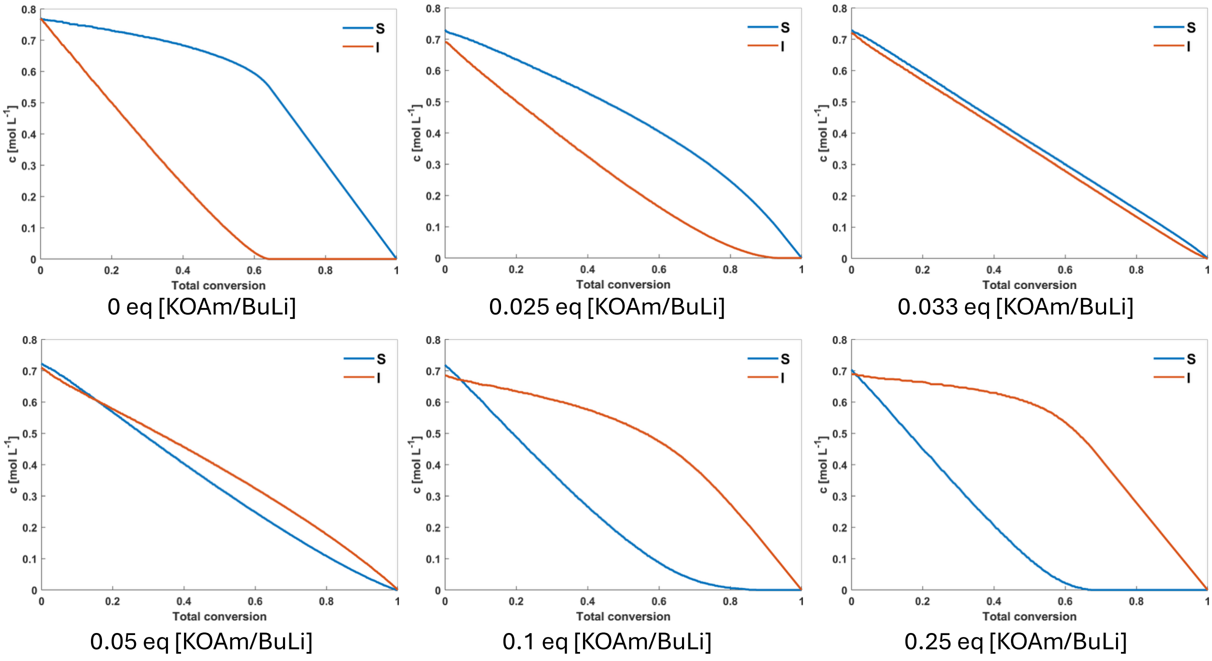

Figure S13: Individual monomer concentrations as a function of total conversion for various [KOAm/BuLi] ratios.

2.4.4 Half-lives of the copolymerizations

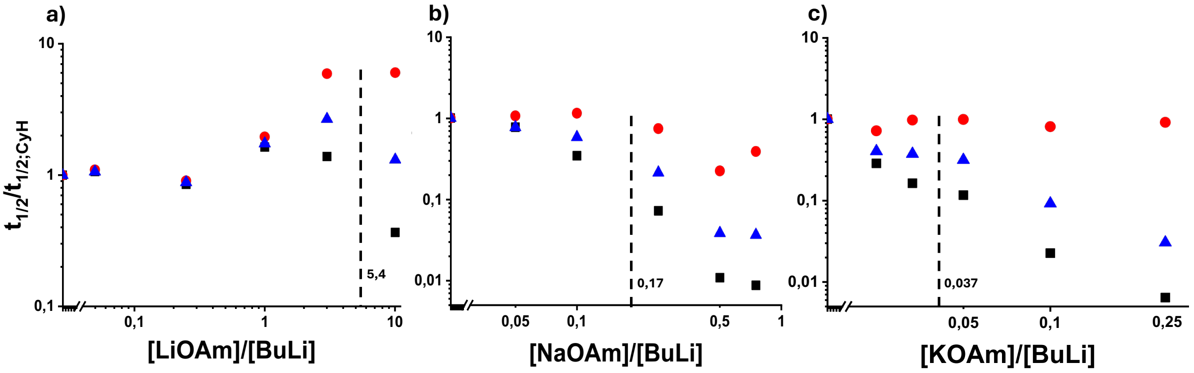

Figure S14: Estimated half-lives of isoprene (red circles), styrene (black squares) and both monomers (blue triangles) in the presence of different amylates. The half lives were normalized with the half-lives in pure cyclohexane, see Table 1.

3. Determination of reactivity ratios:

Reactivity ratios (r_I and r_S) were calculated using the terminal (Jaacks[14,15]) and the non-terminal (Meyer-Lowry[16]) model, which assumes an ideal copolymerization ($r_1 r_2 = 1$). Whenever the linear terminal plot was valid, this model is preferred to avoid overfitting in the non-terminal model.[17]

The reactivity ratios were calculated in the linear range of the individual vs total conversion plots up to 85-90% conversion of the monomer polymerizing faster. The last 5-10% were not taken into account, as these data points are more influenced by unstable baselines and background noises.

3.1 LiOAM

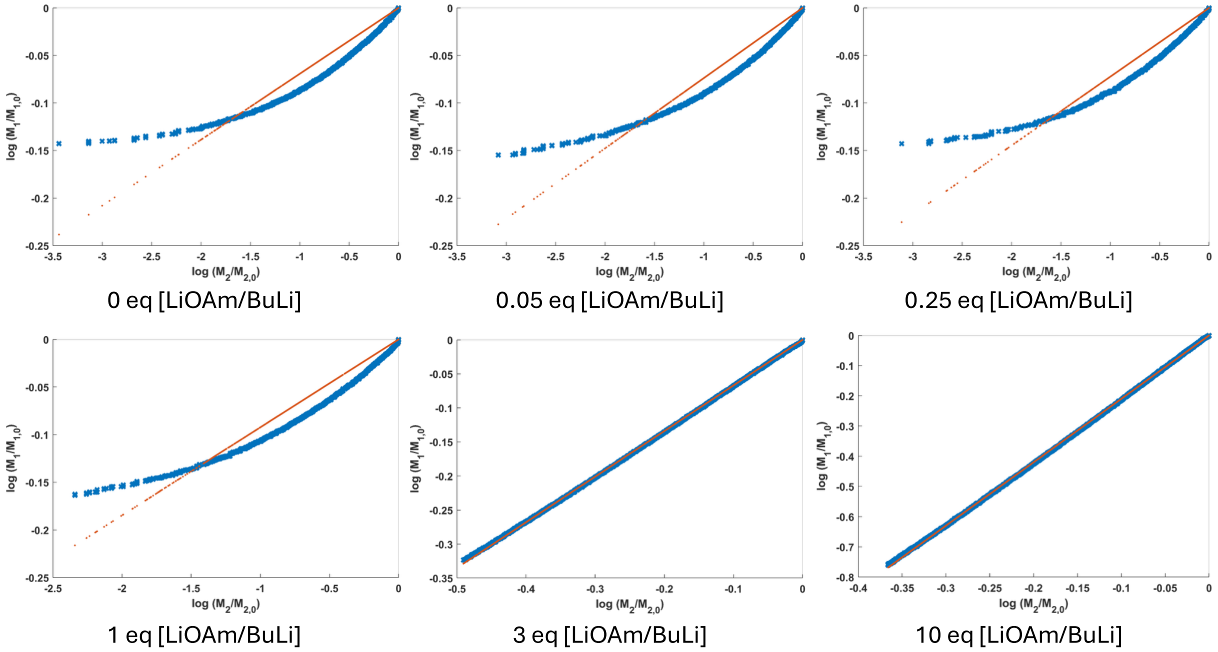

Figure S15: Jaacks fit (red line) of the copolymerization of P($S_{0.5}$-*co*-$I_{0.5}$) with different [LiOAm/BuLi] ratios.

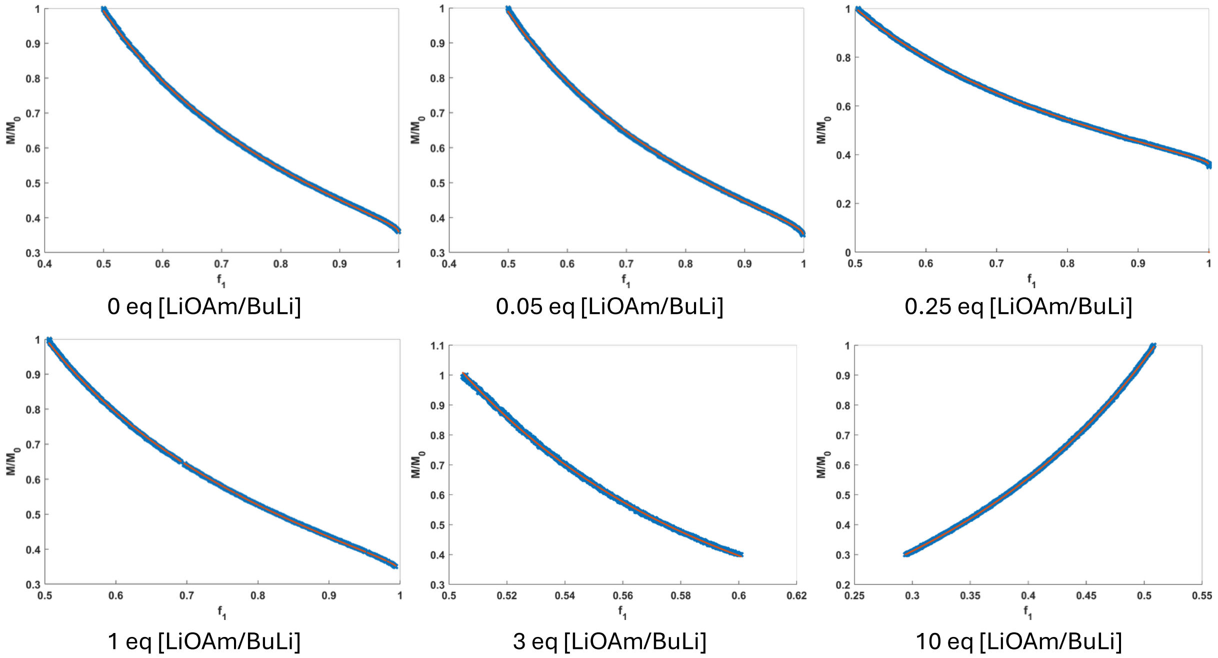

Figure S16: Meyer-Lowry fit (red line) of the copolymerization of P($S_{0.5}$-*co*-$I_{0.5}$) with different [LiOAm/BuLi] ratios.

3.2 NaOAm

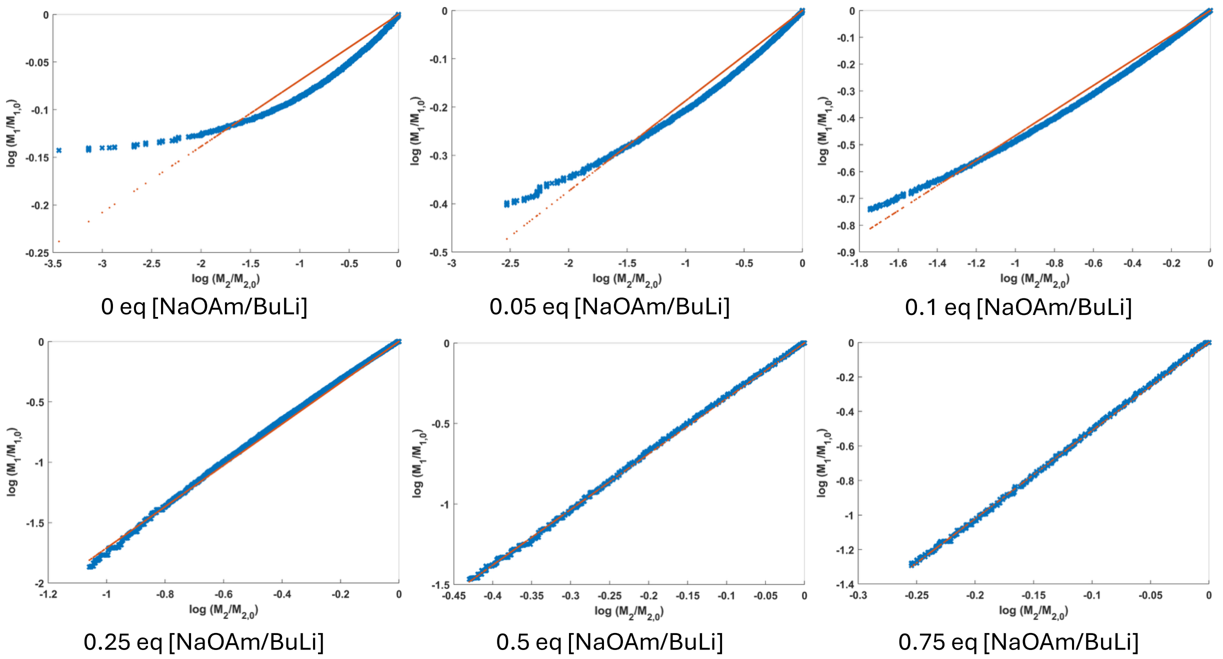

Figure S17: Jaacks fit (red line) of the copolymerization of P($S_{0.5}$-*co*-$I_{0.5}$) with different [NaOAm/BuLi] ratios.

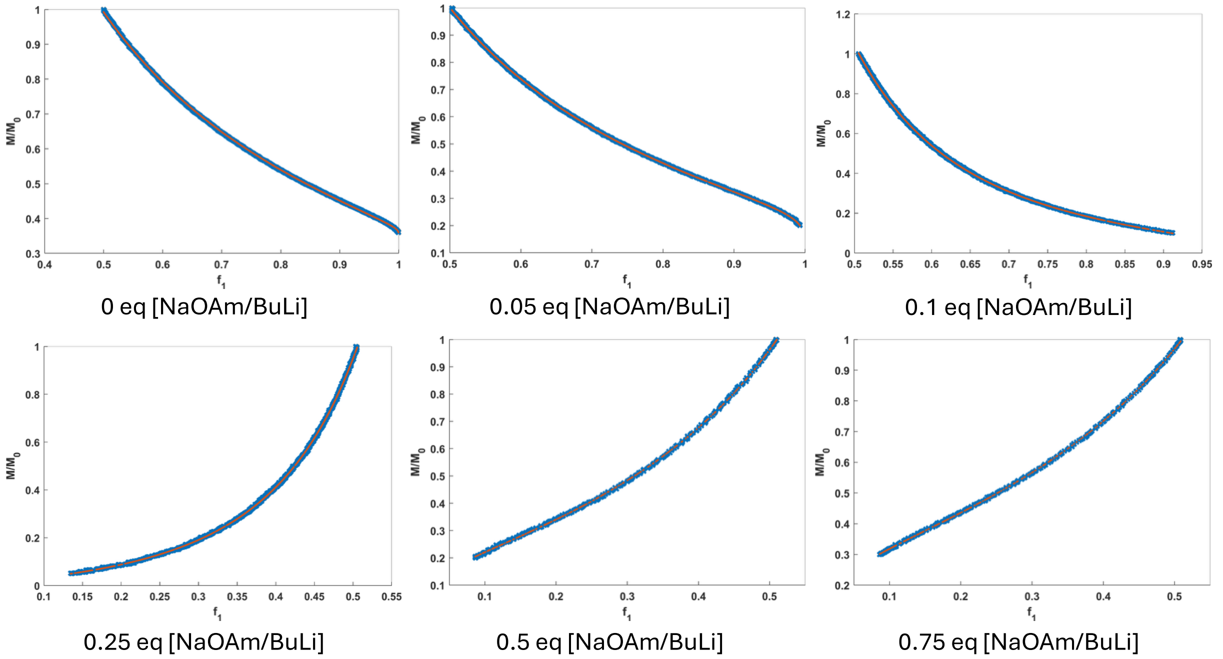

Figure S18 Meyer-Lowry fit (red line) of the copolymerization of P($S_{0.5}$-*co*-$I_{0.5}$) with different [NaOAm/BuLi] ratios.

3.3 KOAM

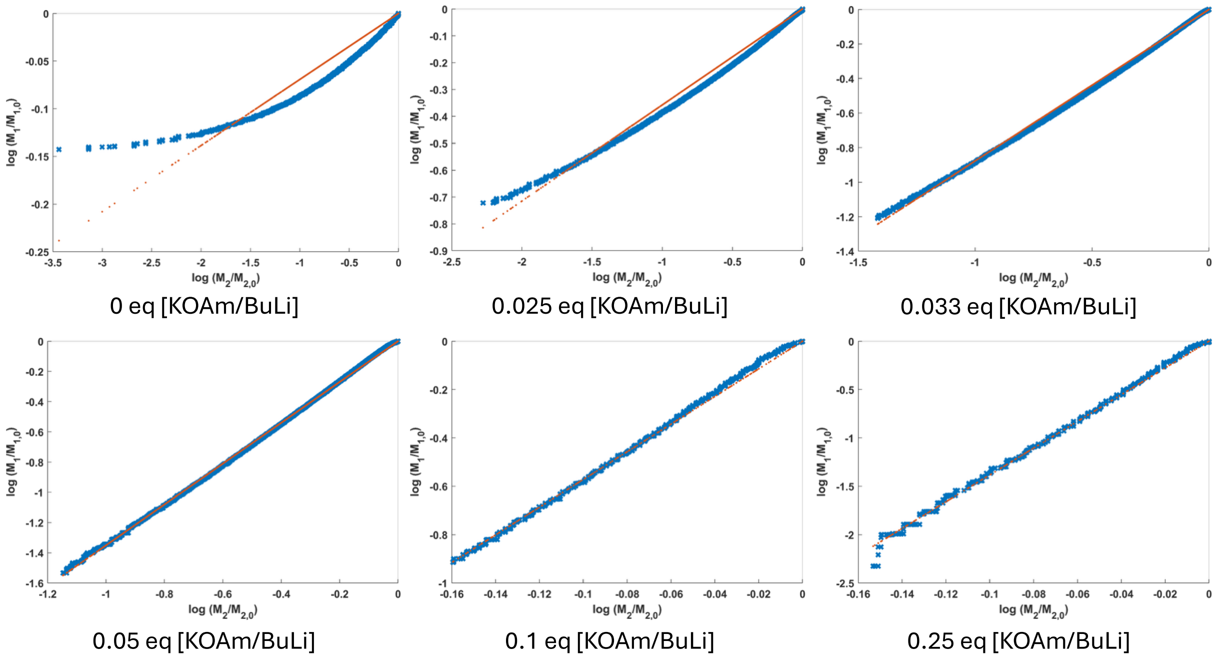

Figure S19: Jaacks fit (red line) of the copolymerization of P($S_{0.5}$-*co*-$I_{0.5}$) with different [KOAm/BuLi] ratios.

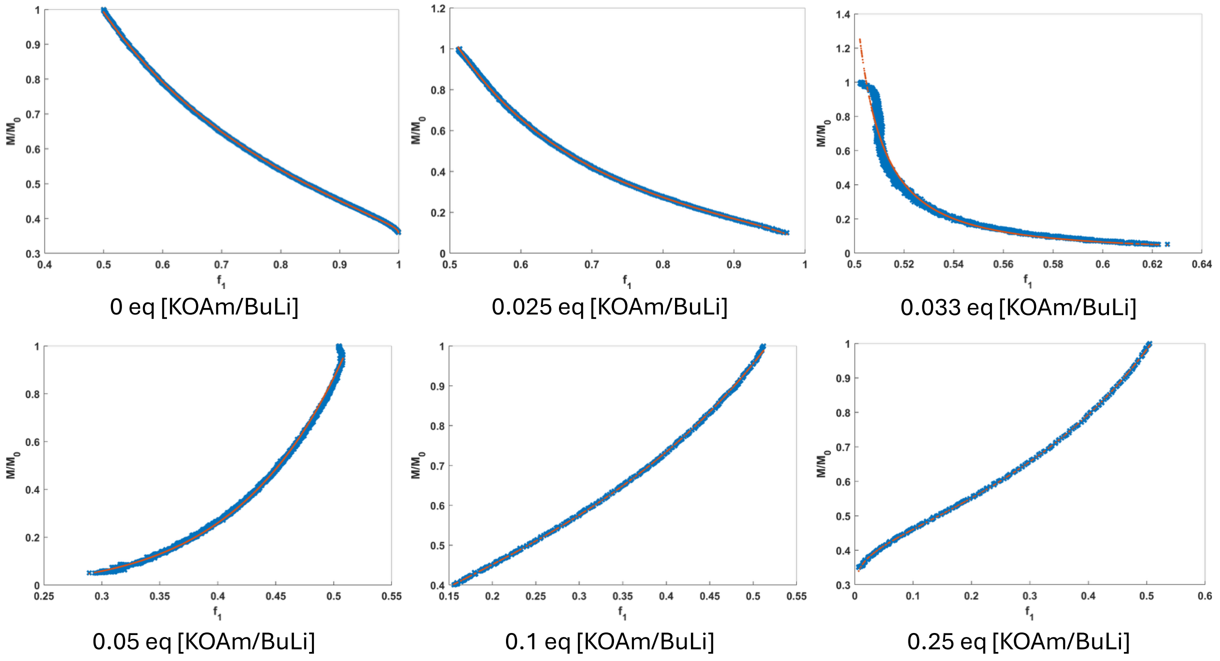

Figure S20: Meyer-Lowry fit (red line) of the copolymerization of P($S_{0.5}$-*co*-$I_{0.5}$) with different [KOAm/BuLi] ratios.

4. Copolymer composition

4.1 Molar and volume composition profiles

The molar copolymer composition were calculated using the reactivity ratios according to Wahlen et al.[18] For the volume based gradients, the monomer molecular weights and densities of the homopolymers, ρ_{PI} = 0.91 g/ml and ρ_{PS} = 1.05 g/ml, were used.

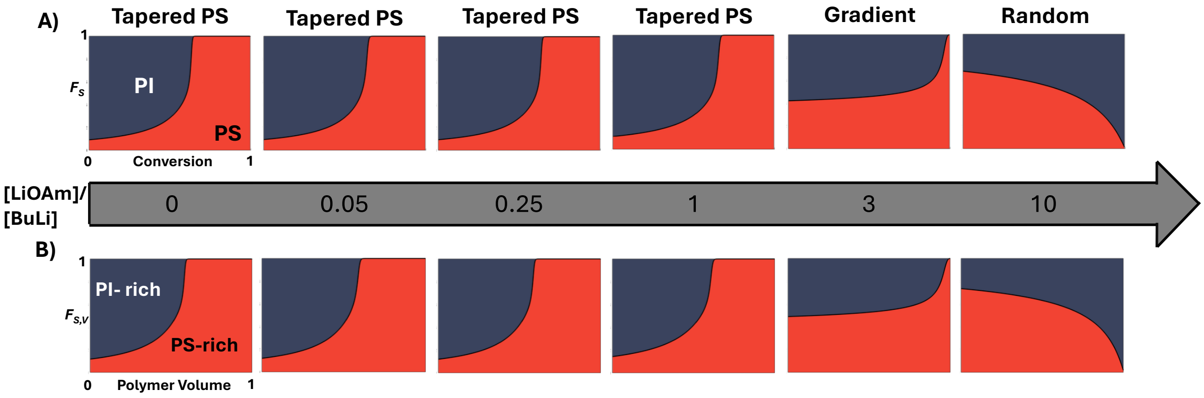

Figure S21: A) Molar B) volume composition profiles of the P($S_{0.5}$-*co*-$I_{0.5}$) copolymer as a function of the [LiOAm]/[BuLi] ratio.

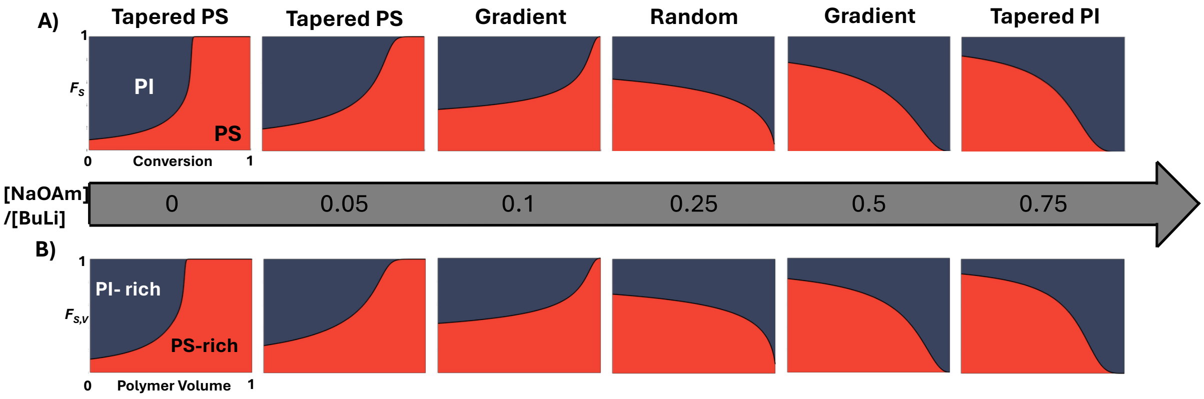

Figure S22: A) Molar B) volume composition profiles of the P($S_{0.5}$-*co*-$I_{0.5}$) copolymer as a function of the [NaOAm]/[BuLi] ratio.

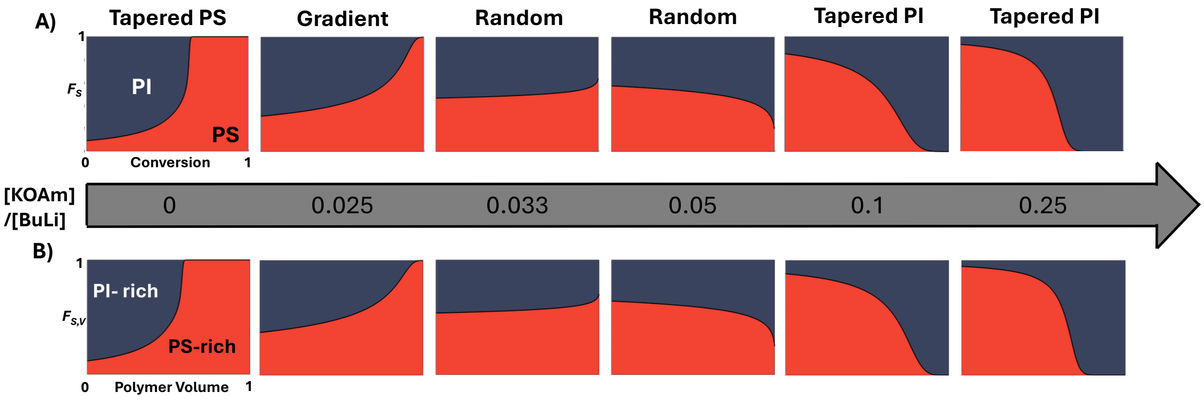

Figure S23: A) Molar B) volume composition profiles of the P($S_{0.5}$-*co*-$I_{0.5}$) copolymer as a function of the [KOAm]/[BuLi] ratio.

4.2 Blockiness

The so-called blockiness of the PS units is defined as the fraction of two to three contiguous styrene units in the copolymer. For block styrene are the ortho protons (h) shifted to 6.93-6.25 ppm in the NMR spectra and with increasing randomness they shift to 7.50-6.93 ppm.[19–22] Further information about this topic can be found in previous publications.[2,3,11,12]

The blockiness was calculated using the integrals of the total aromatic protons: I_1 = 7.50-6.25 ppm (5H) and those of the block styrene, I_2 = 6.93-6.25 ppm (2H):

$$B = \frac{I_2/2}{I_1/5}$$

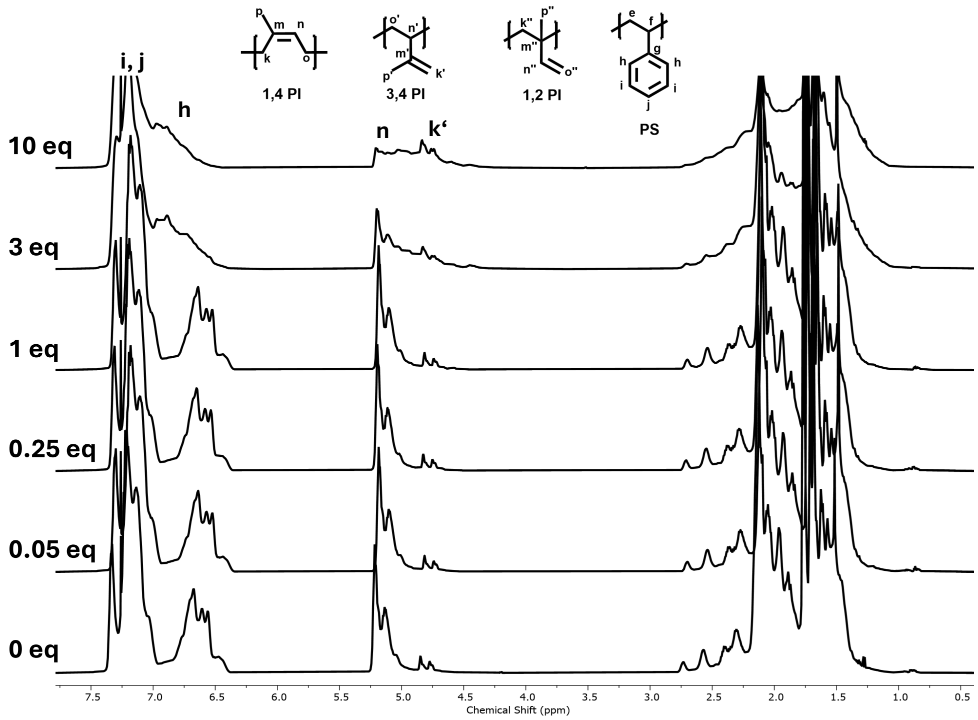

Figure S24: Stacked ^{1}H-NMR spectra of P(S-*co*-I) synthesized with different [LiOAm/BuLi] ratios.

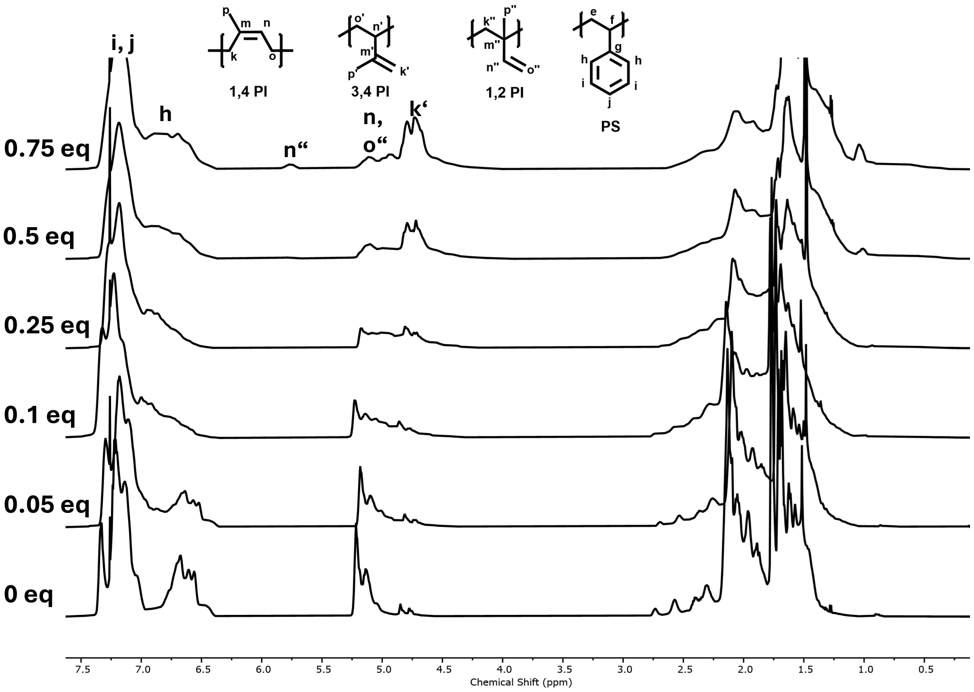

Figure S25 Stacked ^{1}H-NMR spectra of P(S-*co*-I) synthesized with different [NaOAm/BuLi] ratios.

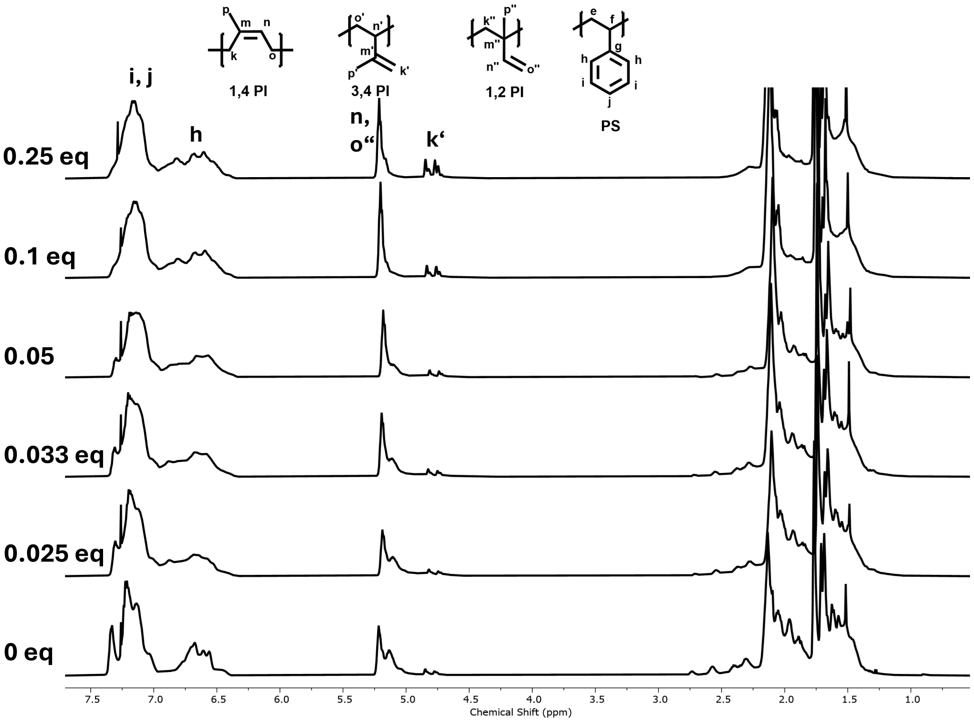

Figure S26 Stacked ^{1}H-NMR spectra of P(S-*co*-I) synthesized with different [KOAm/BuLi] ratios.

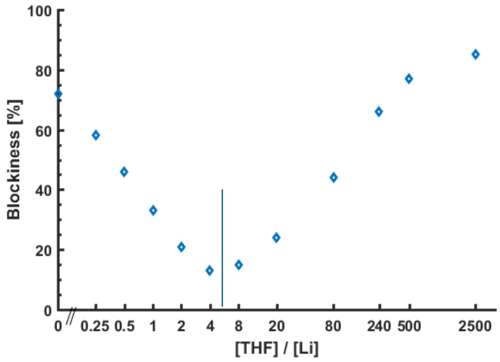

Figure S27. Blockiness of P(S-co-I) as a function of the [THF]/[Li] ratio. The vertical line represents random copolymerization.[3] Reproduced by permit of American Chemical Society.

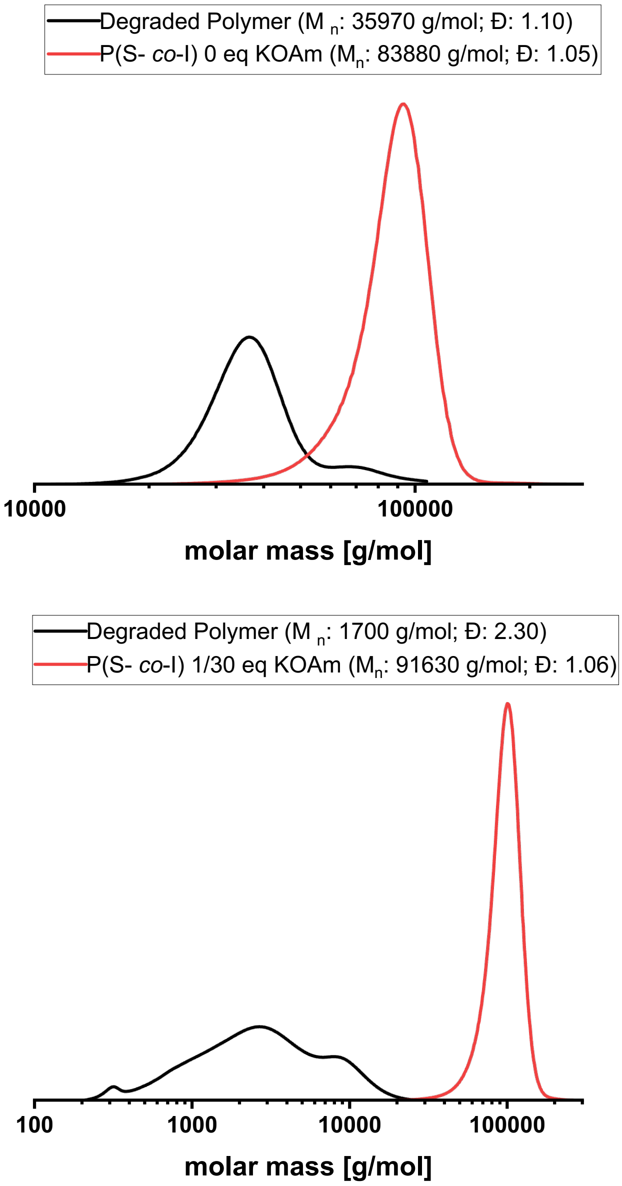

Figure S 28: SEC (THF, PS-calibration) traces of P(S-*co*-I) (blue) synthesized in cyclohexane (top) and in the presence of 1/30 equiv potassium amylate (bottom) and the oxidative degradation product (red).

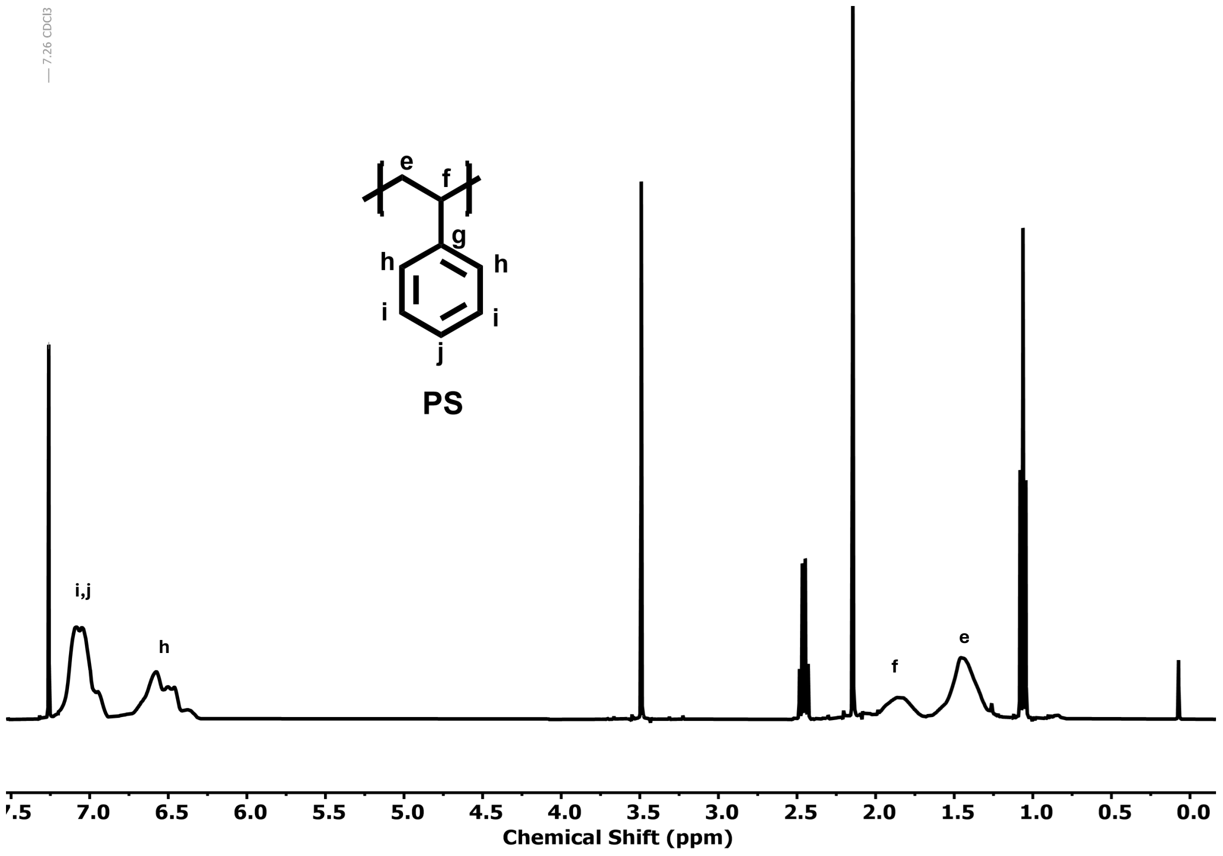

Figure S29: Assigned ^{1}H-NMR spectrum of PS, obtained from degraded P(S-*co*-I) synthesized in cyclohexane with 0.033 equiv KOAm.

5. Microstrcuture of PI units

The microstructure of the PI units was determined via ^{1}H-NMR. With increasing modifier content increase the 3,4- and 1,2-units, which are represented by the following signals: 5.80-5.27 ppm (n''), 4.85-4.38 ppm (k') and 5.30-4.85 ppm (n, o'').

$$I_{1,2} = I(5.80 - 5.27)$$

$$I_{3,4} = I(4.85 - 4.38)$$

$$I_{1,4} = I(5.30 - 4.85) - 2 * I_{1,2}$$

$$C_{1,2} = \frac{I_{1,2}}{I_{1,2} + I_{3,4} + I_{1,4}}$$

$$C_{3,4} = \frac{I_{3,4}}{I_{1,2} + I_{3,4} + I_{1,4}}$$

$$C_{1,4} = \frac{I_{1,4}}{I_{1,2} + I_{3,4} + I_{1,4}}$$

C is the content of the microstructure in the polymer. Partially overlap, especially of the signals k' and n, o'', limits the accuracy of this method. But the results are in good agreement with literature,[1] slight differences can be explained by this overlap, different reaction temperature and different monomer and chain end concentrations.[23]

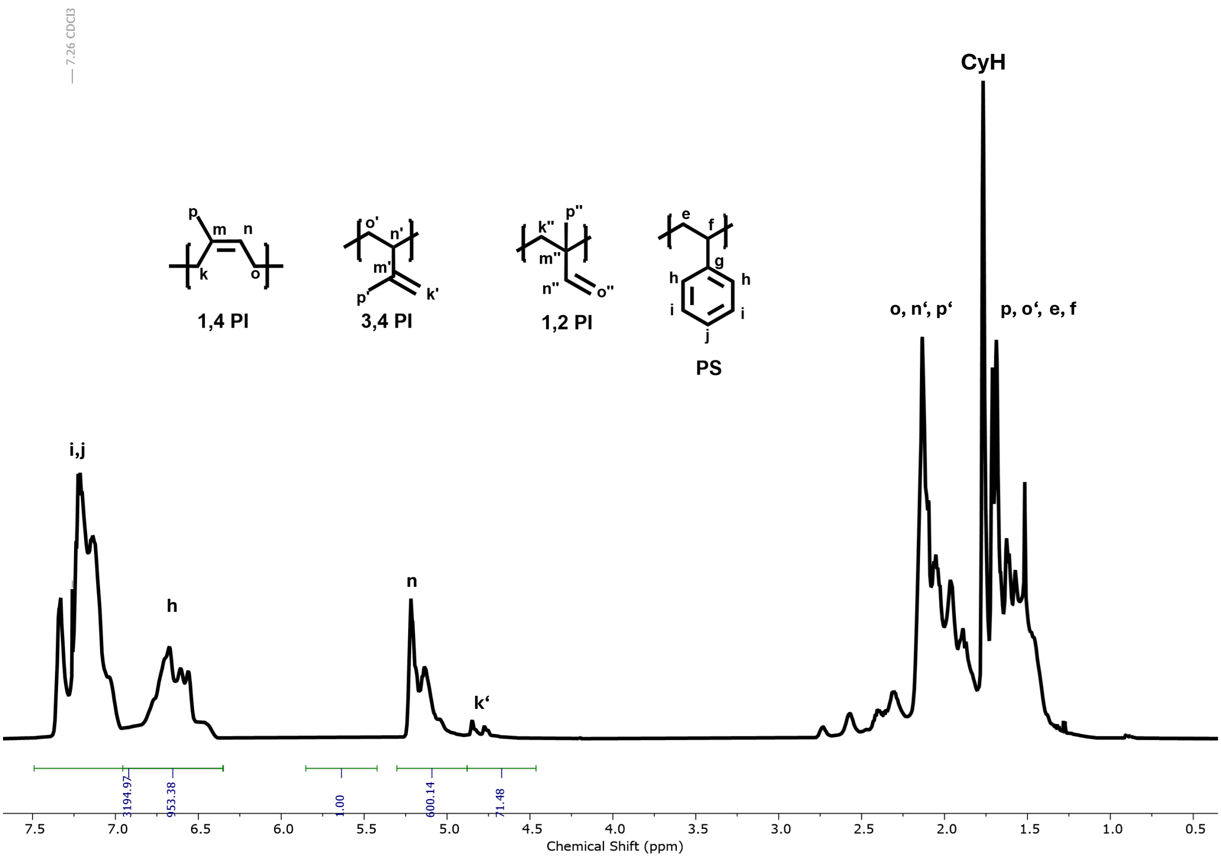

Figure S30: Assigned ^{1}H-NMR spectra of the copolymer P(S-*co*-I) synthesized in pure cyclohexane.

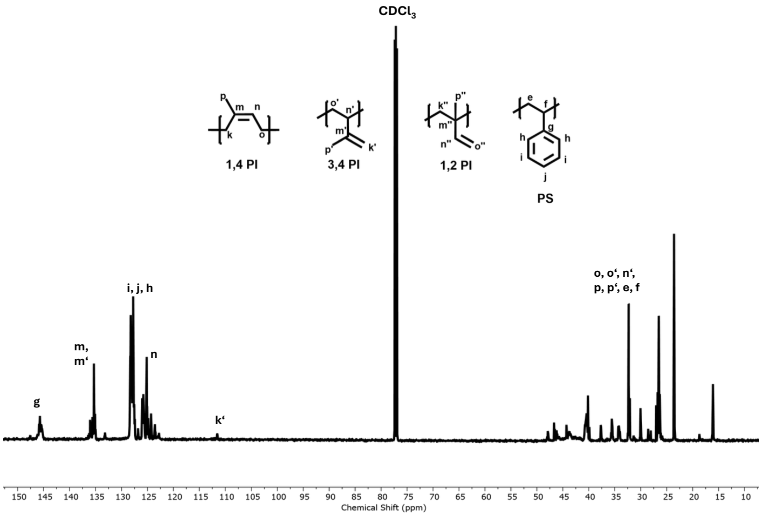

Figure S31: Assigned ^{13}C-NMR spectra of the copolymer P(S-*co*-I) synthesized in pure cyclohexane.

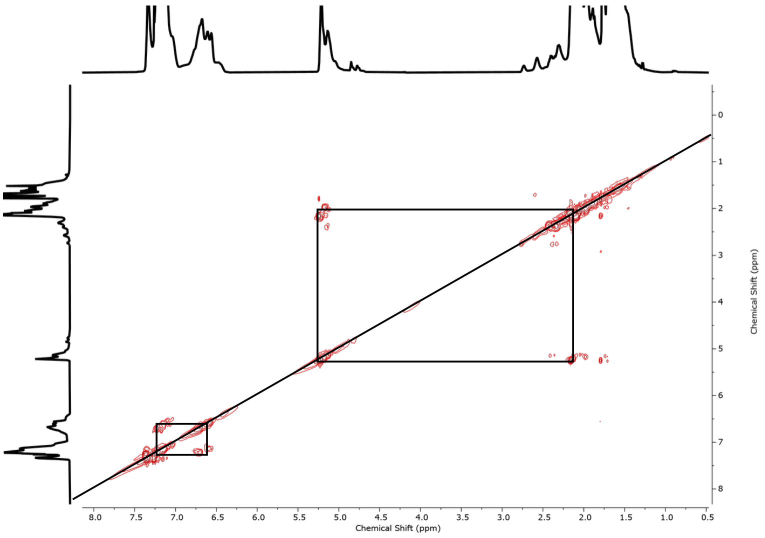

Figure S32: Assigned COSY-NMR spectra of the copolymer P(S-*co*-I) synthesized in pure cyclohexane.

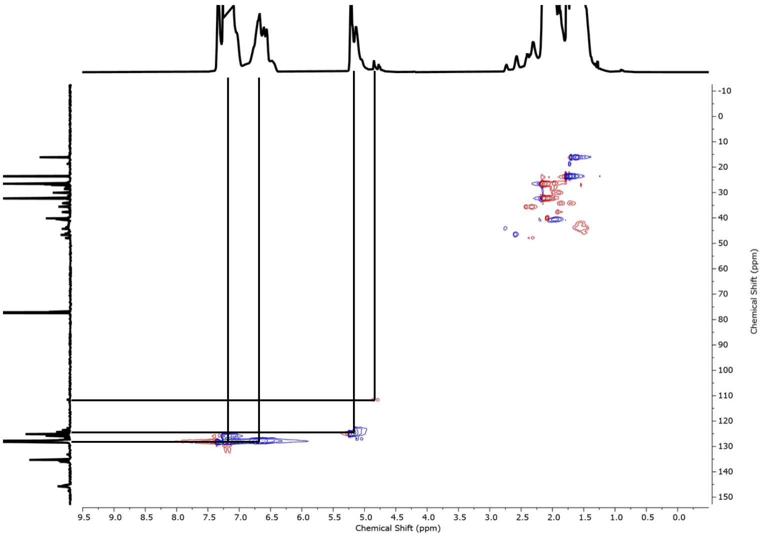

Figure S33: Assigned HSQC-NMR spectra of the copolymer P(S-*co*-I) synthesized in pure cyclohexane.

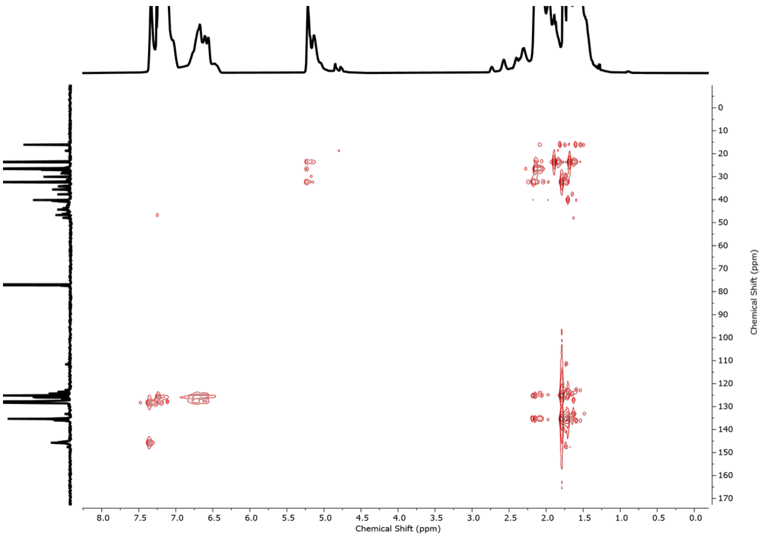

Figure S34: Assigned HMBC-NMR spectra of the copolymer P(S-*co*-I) synthesized in pure cyclohexane.

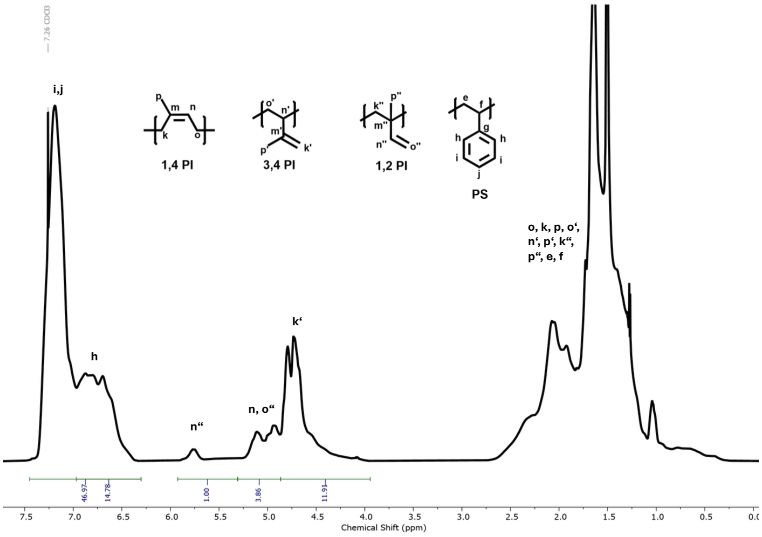

Figure S35: Assigned ^{1}H-NMR spectra of the copolymer P(S-*co*-I) synthesized in cyclohexane with 0.75 equiv. NaOAm.

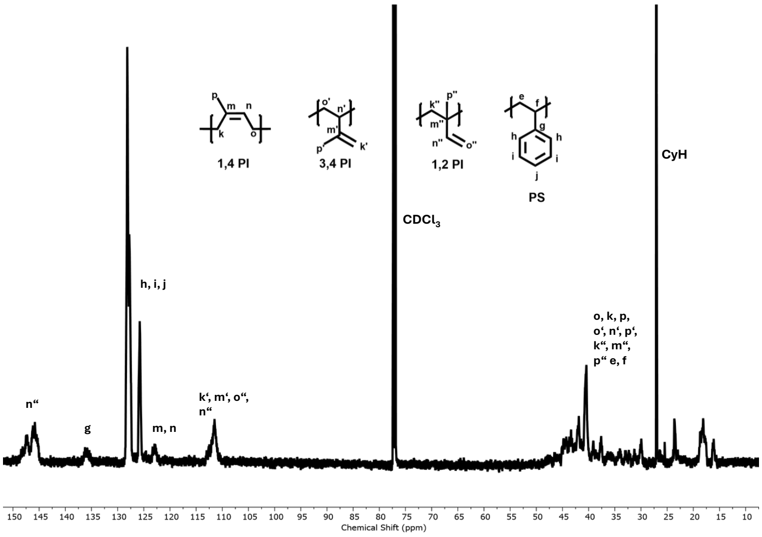

Figure S36: Assigned ^{13}C-NMR spectra of the copolymer P(S-*co*-I) synthesized in cyclohexane with 0.75 equiv. NaOAm.

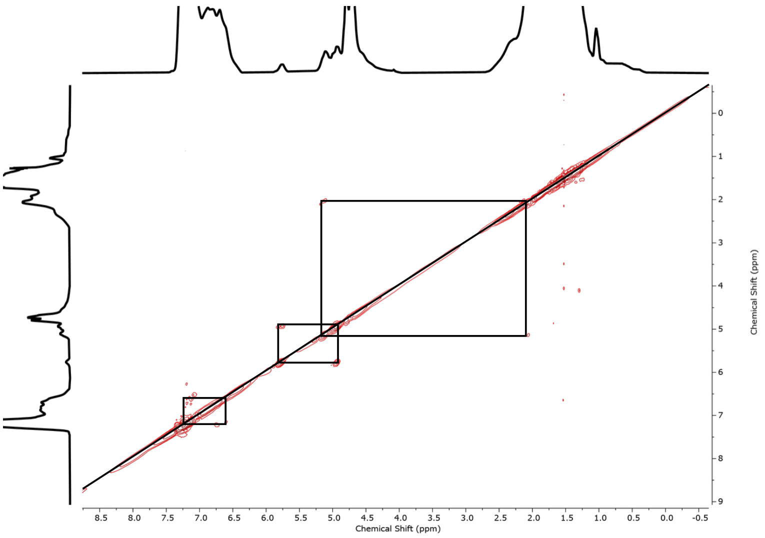

Figure S37: Assigned COSY-NMR spectra of the copolymer P(S-*co*-I) synthesized in cyclohexane with 0.75 equiv. NaOAm.

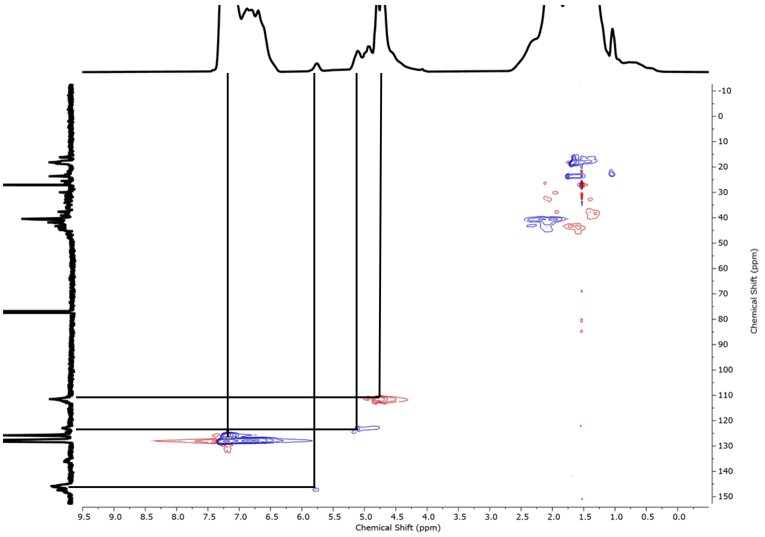

Figure S38: Assigned HSQC-NMR spectra of the copolymer P(S-*co*-I) synthesized in cyclohexane with 0.75 equiv. NaOAm.

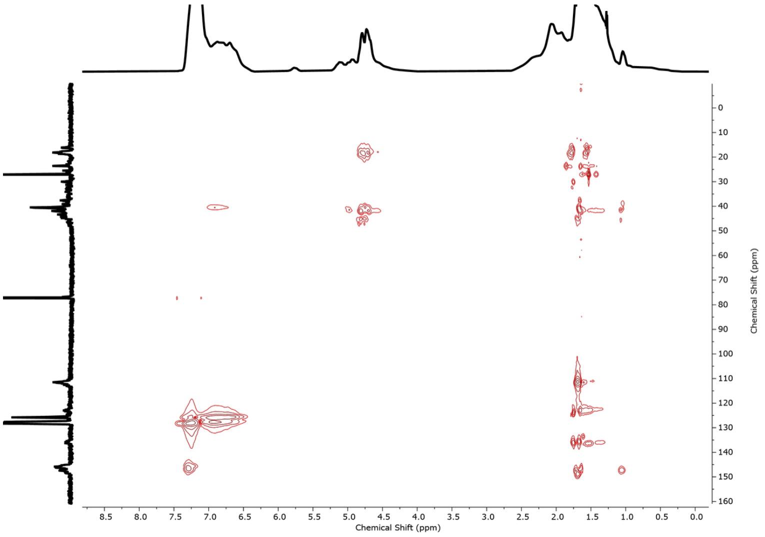

Figure S39: Assigned HMBC-NMR spectra of the copolymer P(S-*co*-I) synthesized in cyclohexane with 0.75 equiv. NaOAm.

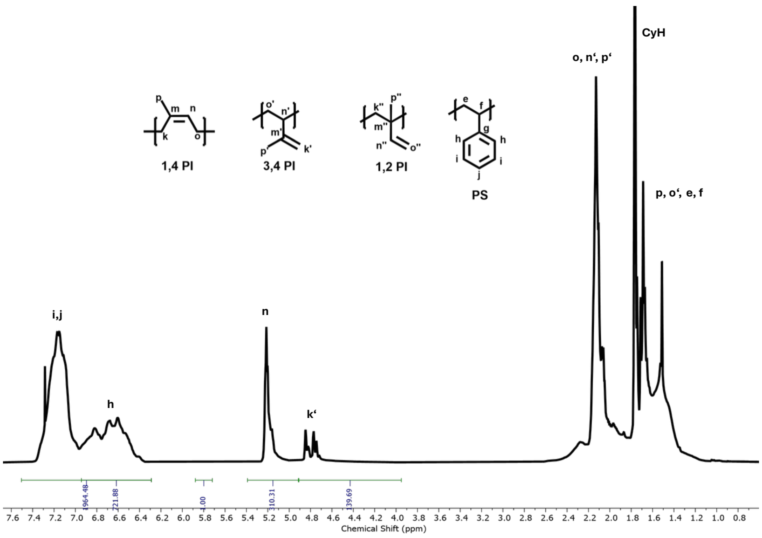

Figure S40: Assigned ^{1}H-NMR spectra of the copolymer P(S-*co*-I) synthesized in cyclohexane with 0.25 equiv. KOAm.

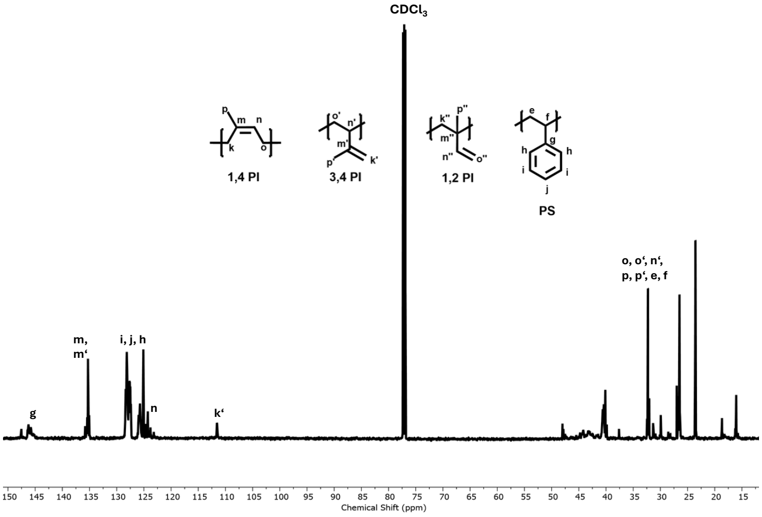

Figure S41: Assigned ^{13}C-NMR spectra of the copolymer P(S-*co*-I) synthesized in cyclohexane with 0.25 equiv. KOAm.

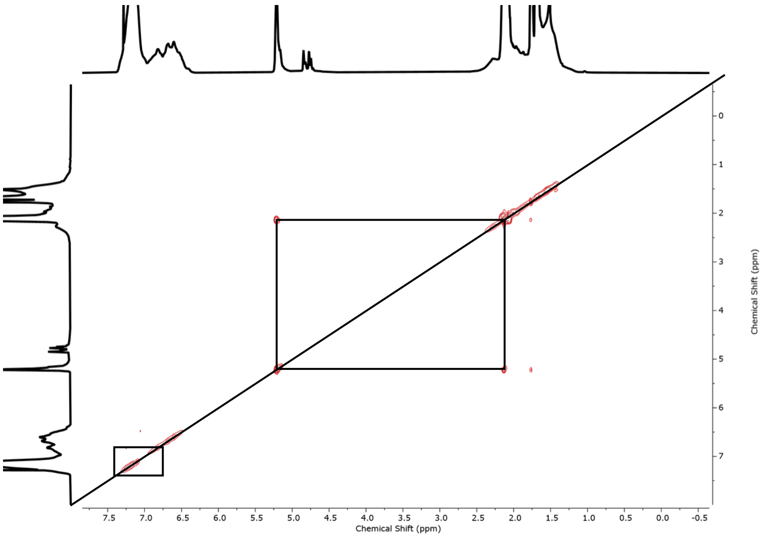

Figure S42: Assigned COSY-NMR spectra of the copolymer P(S-*co*-I) synthesized in cyclohexane with 0.25 equiv. KOAm.

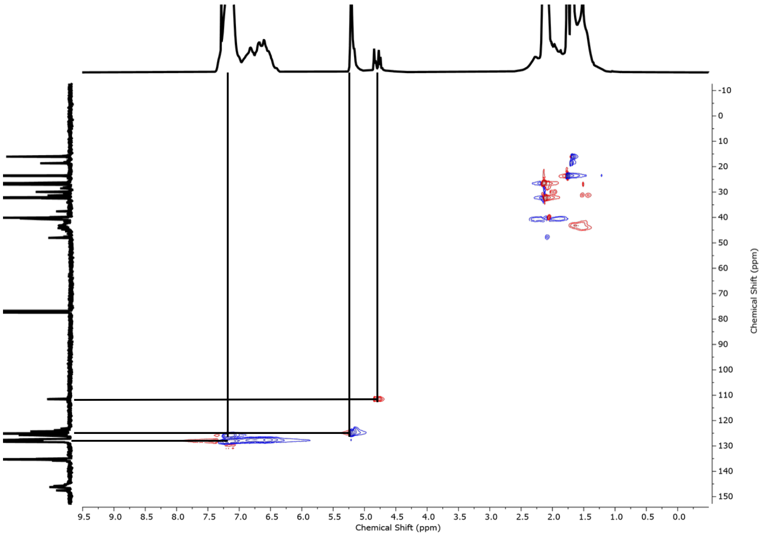

Figure S43: Assigned HSQC-NMR spectra of the copolymer P(S-*co*-I) synthesized in cyclohexane with 0.25 equiv. KOAm.

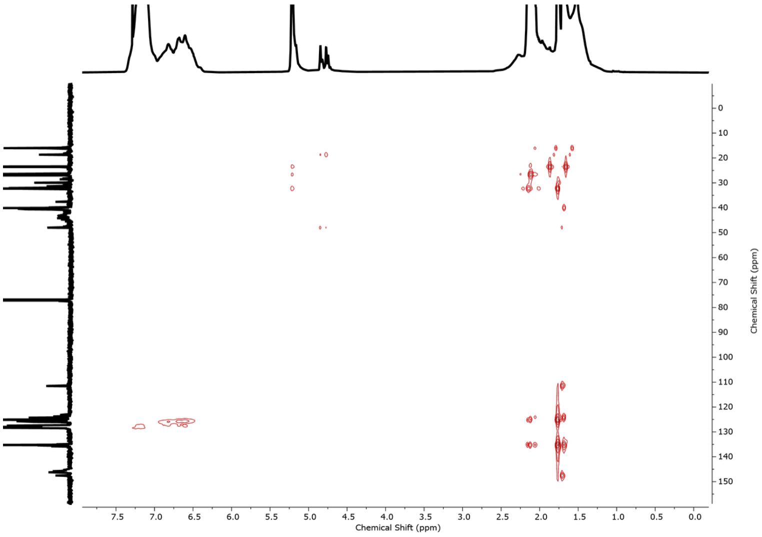

Figure S44: Assigned HMBC-NMR spectra of the copolymer P(S-*co*-I) synthesized in cyclohexane with 0.25 equiv. KOAm.

6. Glass transition temperatures

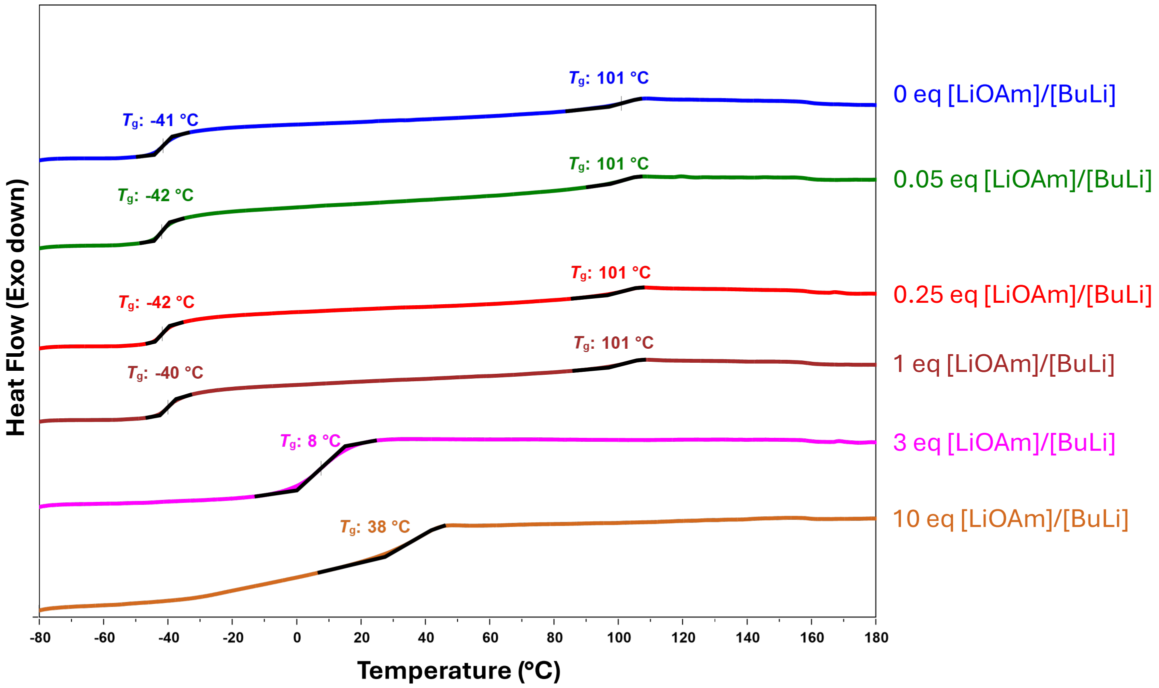

Figure S45: Second DSC heating curve of P(S-*co*-I) synthesized with different amounts of LiOAm, measured with a heating rate of 10 K min^{-1}.

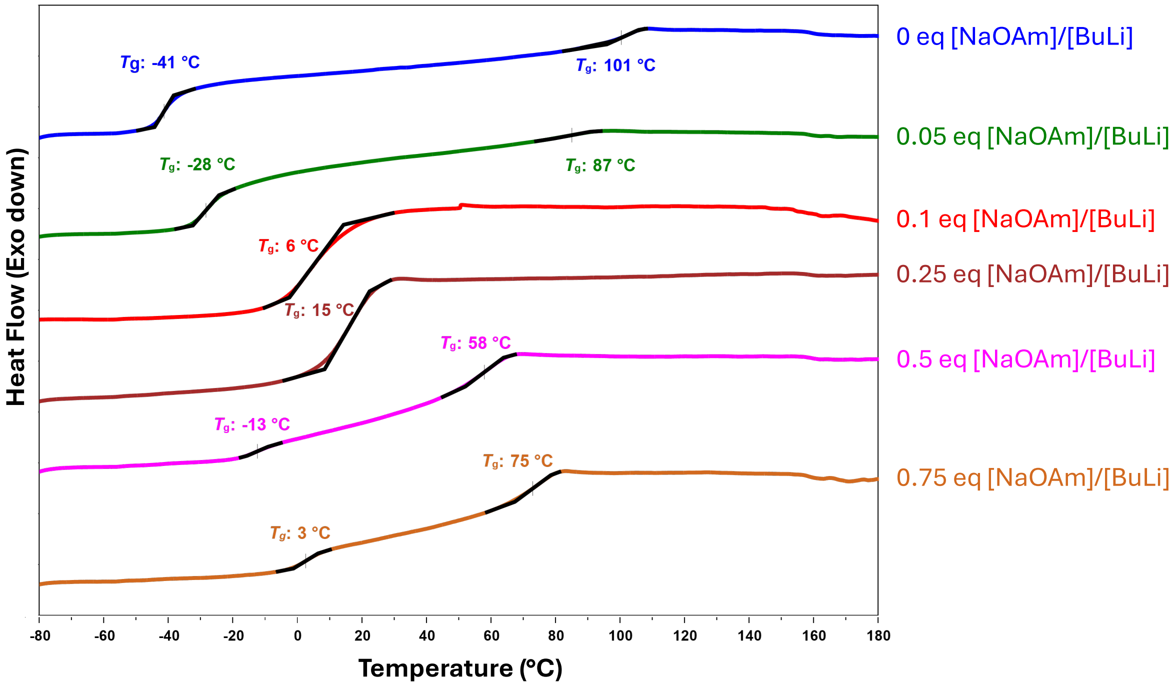

Figure S46: Second DSC heating curve of P(S-*co*-I) synthesized with different amounts of NaOAm, measured with a heating rate of 10 K min^{-1}.

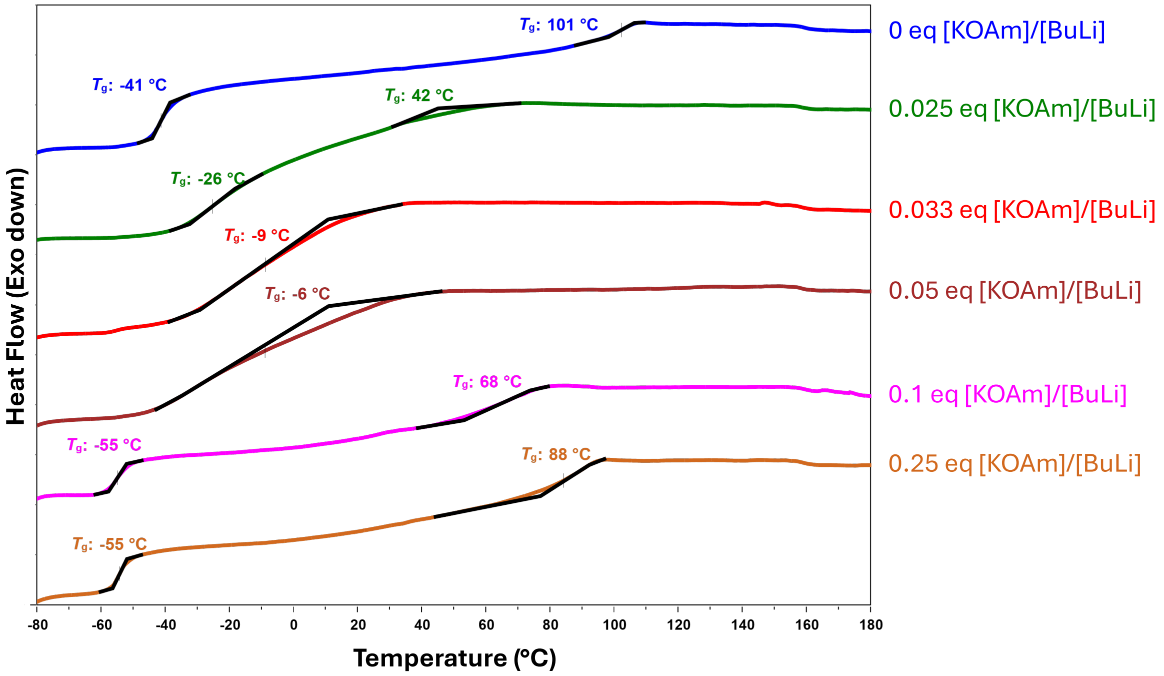

Figure S47: Second DSC heating curve of P(S-*co*-I) synthesized with different amounts of KOAm, measured with a heating rate of 10 K min^{-1}.

7. References

(1) Steube, M.; Johann, T.; Plank, M.; Tjaberings, S.; Gröschel, A. H.; Gallei, M.; Frey, H.; Müller, A. H. E. Kinetics of Anionic Living Copolymerization of Isoprene and Styrene Using in Situ NIR Spectroscopy: Temperature Effects on Monomer Sequence and Morphology. *Macromolecules* **2019**, *52* (23), 9299–9310. https://doi.org/10.1021/acs.macromol.9b01790.

(2) Fuchs, D. A. H.; Hübner, H.; Kraus, T.; Niebuur, B.-J.; Gallei, M.; Frey, H.; Müller, A. H. E. The Effect of THF and the Chelating Modifier DTHFP on the Copolymerisation of β-Myrcene and Styrene: Kinetics, Microstructures, Morphologies, and Mechanical Properties. *Polym Chem* **2021**, *12* (32), 4632–4642. https://doi.org/10.1039/D1PY00791B.

(3) Steube, M.; Johann, T.; Hübner, H.; Koch, M.; Dinh, T.; Gallei, M.; Floudas, G.; Frey, H.; Müller, A. H. E. Tetrahydrofuran: More than a "Randomizer" in the Living Anionic Copolymerization of Styrene and Isoprene: Kinetics, Microstructures, Morphologies, and Mechanical Properties. *Macromolecules* **2020**, *53* (13), 5512–5527. https://doi.org/10.1021/acs.macromol.0c01022.

(4) Schlosser, M. Superbases for Organic Synthesis. *Pure and Applied Chemistry* **1988**, *60* (11), 1627–1634. https://doi.org/10.1351/pac198860111627.

(5) Lochmann, L.; Janata, M. 50 Years of Superbases Made from Organolithium Compounds and Heavier Alkali Metal Alkoxides. *Open Chem* **2014**, *12* (5), 537–548. https://doi.org/10.2478/s11532-014-0528-0.

(6) Forens, A.; Roos, K.; Dire, C.; Gadenne, B.; Carlotti, S. Anionic Polymerization of Butadiene Using Lithium/Potassium Multi-Metallic Systems: Influence on Polymerization

Control and Polybutadiene Microstructure. *Chinese Journal of Polymer Science (English Edition)* **2020**, *38* (4), 357–362. https://doi.org/10.1007/s10118-020-2355-4.

(7) Lochmann, L.; Pospíšil, J.; Lím, D. On the Interaction of Organolithium Compounds with Sodium and Potassium Alkoxides. A New Method for the Synthesis of Organosodium and Organopotassium Compounds. *Tetrahedron Lett* **1966**, *7* (2), 257–262. https://doi.org/10.1016/S0040-4039(00)70224-3.

(8) Kirchevskaya, I. Yu.; Samotsvetov, A. R.; Seredina, N. P.; Urazov, N. I.; Shatalov, V. P. Relations between Polymerization of Hydrocarbon Monomers in the Presence of Lithium Alkyls Modified with Potassium Tert.Butylate. *Polymer Science U.S.S.R.* **1976**, *18* (8), 2111–2118. https://doi.org/10.1016/0032-3950(76)90398-1.

(9) Forens, A.; Roos, K.; Dire, C.; Gadenne, B.; Carlotti, S. Anionic Polymerization of Butadiene Using Lithium/Potassium Multi-Metallic Systems: Influence on Polymerization Control and Polybutadiene Microstructure. *Chinese Journal of Polymer Science (English Edition)* **2020**, *38* (4), 357–362. https://doi.org/10.1007/s10118-020-2355-4.

(10) Halasa, A. F.; Mitchell, G. B.; Stayer, M.; Tate, D. P.; Oberster, A. E.; Koch, R. W. Metalation of Unsaturated Polymers by Using Activated Organolithium Compounds and the Formation of Graft Copolymers. II. *Journal of Polymer Science: Polymer Chemistry Edition* **1976**, *14* (2), 497–506. https://doi.org/10.1002/pol.1976.170140220.

(11) Fuchs, D. A. H.; Wadgaonkar, S. P.; Müller, A. H. E.; Frey, H. Effect of <scp>tetrahydrofuran</Scp> on the Anionic Copolymerization of 4-trimethylsilylstyrene with Isoprene. *Polym Adv Technol* **2024**, *35* (6). https://doi.org/10.1002/pat.6478.

(12) Meier-Merziger, M.; Fuchs, D. A. H.; Frey, H.; Müller, A. H. E. Spotlight on Methyl Tert-Butyl Ether–Underrated or Overlooked? Unveiling Its Role for Living Anionic Polymerization. *Macromolecules* **2024**. https://doi.org/10.1021/acs.macromol.4c01262.

(13) Corbin, N.; Prud'homme, J. MULTIBLOCK COPOLYMERS OF STYRENE AND ISOPRENE - 1. SYNTHESIS AND CHARACTERIZATION. *J Polym Sci Polym Chem Ed* **1976**, *14* (7), 1645–1659. https://doi.org/10.1002/pol.1976.170140708.

(14) Jaacks, V. Eine Neuartige Methode Zur Bestimmung von Copolymerisationsparametern. *Angewandte Chemie* **1967**, *79* (9), 419–419. https://doi.org/10.1002/ange.19670790927.

(15) Jaacks, V. A Novel Method of Determination of Reactivity Ratios in Binary and Ternary Copolymerizations. *Makromol Chem* **1972**, *161* (1), 161–172. https://doi.org/10.1002/macp.1972.021610110.

(16) Meyer, V. E.; Lowry, G. G. Integral and Differential Binary Copolymerization Equations. *J Polym Sci A* **1965**, *3* (8), 2843–2851. https://doi.org/10.1002/pol.1965.100030811.

(17) Hawkins, D. M. The Problem of Overfitting. *J Chem Inf Comput Sci* **2004**, *44* (1), 1–12. https://doi.org/10.1021/ci0342472.

(18) Wahlen, C.; Blankenburg, J.; Von Tiedemann, P.; Ewald, J.; Sajkiewicz, P.; Müller, A. H. E.; Floudas, G.; Frey, H. Tapered Multiblock Copolymers Based on Farnesene and Styrene: Impact of Biobased Polydiene Architectures on Material Properties. *Macromolecules* **2020**, *53* (23), 10397–10408. https://doi.org/10.1021/acs.macromol.0c02118.

(19) Handlin, D. L.; Williamson, D. T.; Willis, C. L. US 6,699,941 B1 Block Copolymer, 2004.

(20) Mochel, V. D. NMR Composition Analysis of Copolymers. *Rubber Chem Technol* **1967**, *40* (4), 1200–1211. https://doi.org/10.5254/1.3539131.

(21) Hogan, T. E.; Kiridena, W.; Kocsis, L. Effect of Stereochemistry in Anionic Polymerization Modifiers. *Rubber Chemistry and Technology* **2017**, *90* (2), 325–336. https://doi.org/10.5254/rct.17.82692.

(22) Sardelis, K.; Michels, H. J.; Allen, G.; F.R.S. Graded Block and Randomized Copolymers of Butadiene-Styrene. *Polymer (Guildf)* **1984**, *25* (7), 1011–1019. https://doi.org/10.1016/0032-3861(84)90089-2.

(23) Uraneck, C. A. Influence of Temperature on Microstructure of Anionic-initiated Polybutadiene. *J Polym Sci A1* **1971**, *9* (8), 2273–2281. https://doi.org/10.1002/pol.1971.150090814.
